# A 50‐year perspective on the use and potential of artiodactyl calcanei in bone adaptation studies

**DOI:** 10.1111/brv.70089

**Published:** 2025-10-28

**Authors:** John G. Skedros

**Affiliations:** ^1^ Bone and Joint Research Laboratory 5316 South Woodrow Street, Ste 200 Salt Lake City UT 84107 USA; ^2^ Department of Orthopaedic Surgery University of Utah 590 Wakara Way Salt Lake City UT 84108 USA

**Keywords:** bone adaptation, deer calcaneus, sheep calcaneus, osteonal remodelling, collagen fibre orientation, strain mode, human femoral neck

## Abstract

Sheep and deer calcanei are important models for studying cortical (compact) and trabecular (cancellous) bone adaptation because they are amenable to direct strain measurement (due to lack of surrounding muscles), experience relatively simple/unidirectional bending, exhibit osteon remodelling, and have the most pronounced regional variations in mineralization and other histological characteristics reported in any bone. This simple loading environment is characterized by bending that produces prevalent/predominant tension on the plantar side and predominant compression on the dorsal side of the cantilevered, beam‐like shaft of these bones. Histocompositional differences are clear between these opposing cortices, optimizing their mechanical properties for these regional differences in loading environment. This keeps the fracture risk low by enhancing the safety factor of the entire bone. Understanding how mechanosensitive cells within bone accomplish this is fundamentally important for advancing core concepts in bone biology and functional adaptation, and for clinical applications. However, an uncontested 1995 study used qualitative histological observations from a small sample (two sub‐adults; three adults) of domesticated sheep calcanei and *in vivo* strain data from Lanyon's seminal study of sheep calcanei to reject the idea that this bone is simply loaded. That study argued that reversals of bending during the swing phase of gait negate the ‘tension/compression (plantar/dorsal)’ concept, thus invalidating much of the basic and translational value of the model. Their opinion is important because many investigators consider it valid despite contrary conclusions of subsequent biomechanical/histomorphological studies. This review critically evaluates the foundations of the main conclusion of that 1995 study, because their refutation of the simplicity of the artiodactyl calcaneus model has been favourably cited nearly 60 times in the peer‐reviewed literature. After exposing and correcting errors and reconciling contradictory observations in that study, this review explores the strengths, limitations, and potential applications of the artiodactyl calcaneus model for advancing understanding of mechanisms and consequences of bone adaptation. Studies reviewed herein support viewing artiodactyl calcanei as simply loaded ‘tension/compression bones’, validating their continued use in this context in a broad spectrum of studies of cortical and trabecular bone adaptation. A particularly promising application of this model is that it can serve as a ‘control bone’ for studies of other presumably simply loaded bone regions, such as the human femoral neck, especially regarding the relationship of its load history, structural and material organization, and propensity to fracture.

## I. INTRODUCTION

**Fig. 1:**
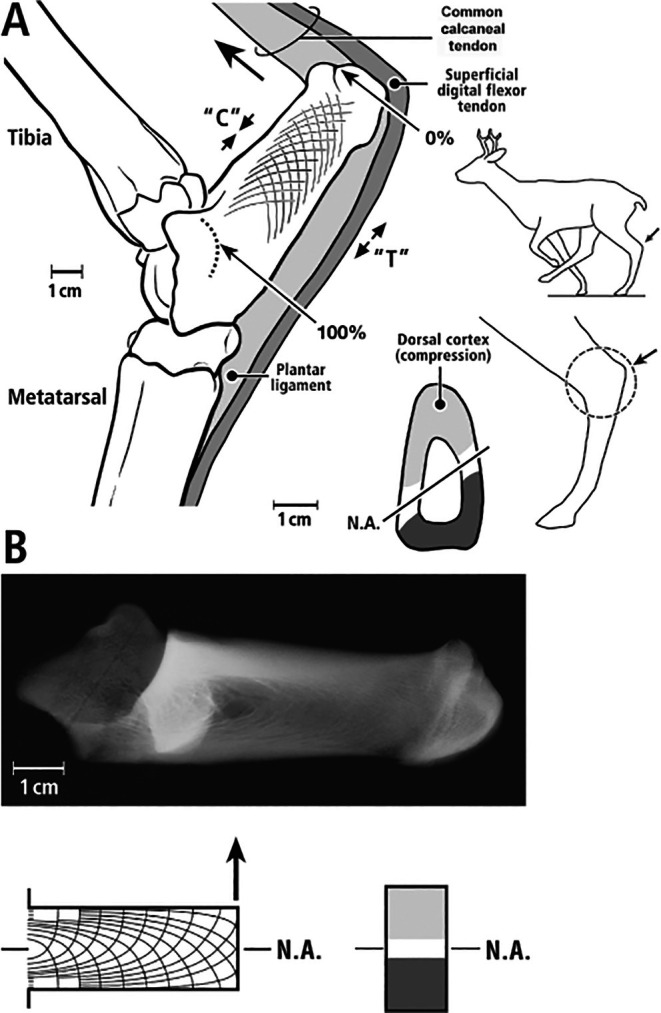
(A) Lateral view of left adult mule deer and the ankle (hock) region (circled in leg image on right). Small arrows indicate the distal portion of the calcaneus in both the full‐body outline and the enlarged drawing of the ankle region. The large arrow in the enlarged drawing shows the direction of force applied to the calcaneus by the common calcaneal (Achilles) tendon during weight bearing, thus loading the dorsal cortex in dominant compression (C) and plantar cortex in dominant tension (T). In the cross‐section on the right, the position of the neutral axis (N.A.) is indicated. (B) Lateral radiograph (top) of an adult mule deer calcaneus showing arched trabecular patterns that resemble the theoretical stress trajectories depicted by the curved lines that form arched patterns in a short, end‐loaded cantilevered beam undergoing bending (bottom). On the bottom right, a transverse section of the beam shows its rectangular shape and horizontal neutral axis, as expected in an idealized cantilevered beam (i.e. orthogonally end‐loaded, prismatic shape, and made of uniform, homogeneous, and isotropic material). Reproduced with some modifications from Skedros *et al*. (2012b, 2019, 2023) with permission of John G. Skedros.

**Fig. 2:**
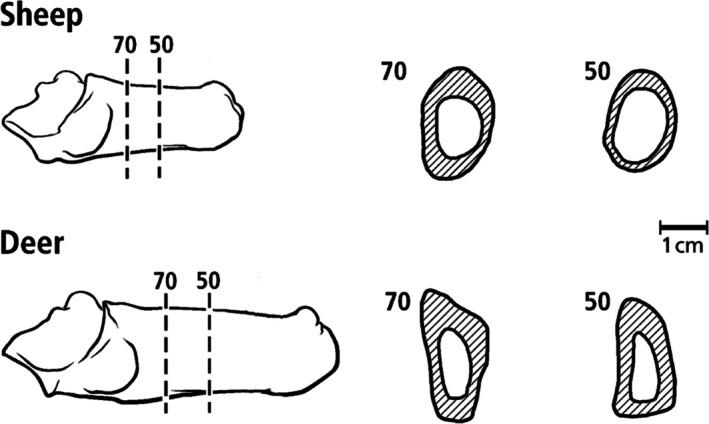
Medial views of adult sheep (top) and mule deer (bottom) calcanei, and transverse sections. The vertical lines indicate the 50 and 70% shaft‐length locations commonly used for histomorphological analysis. These locations are in the more simply loaded portion of the calcaneal shaft; loading of the distal shaft is believed to be more complex (Skedros *et al*., 1994a). The scale bar refers to the cross sections. Reproduced from Sinclair *et al*. (2013) with permission of John G. Skedros.

**Fig. 3:**
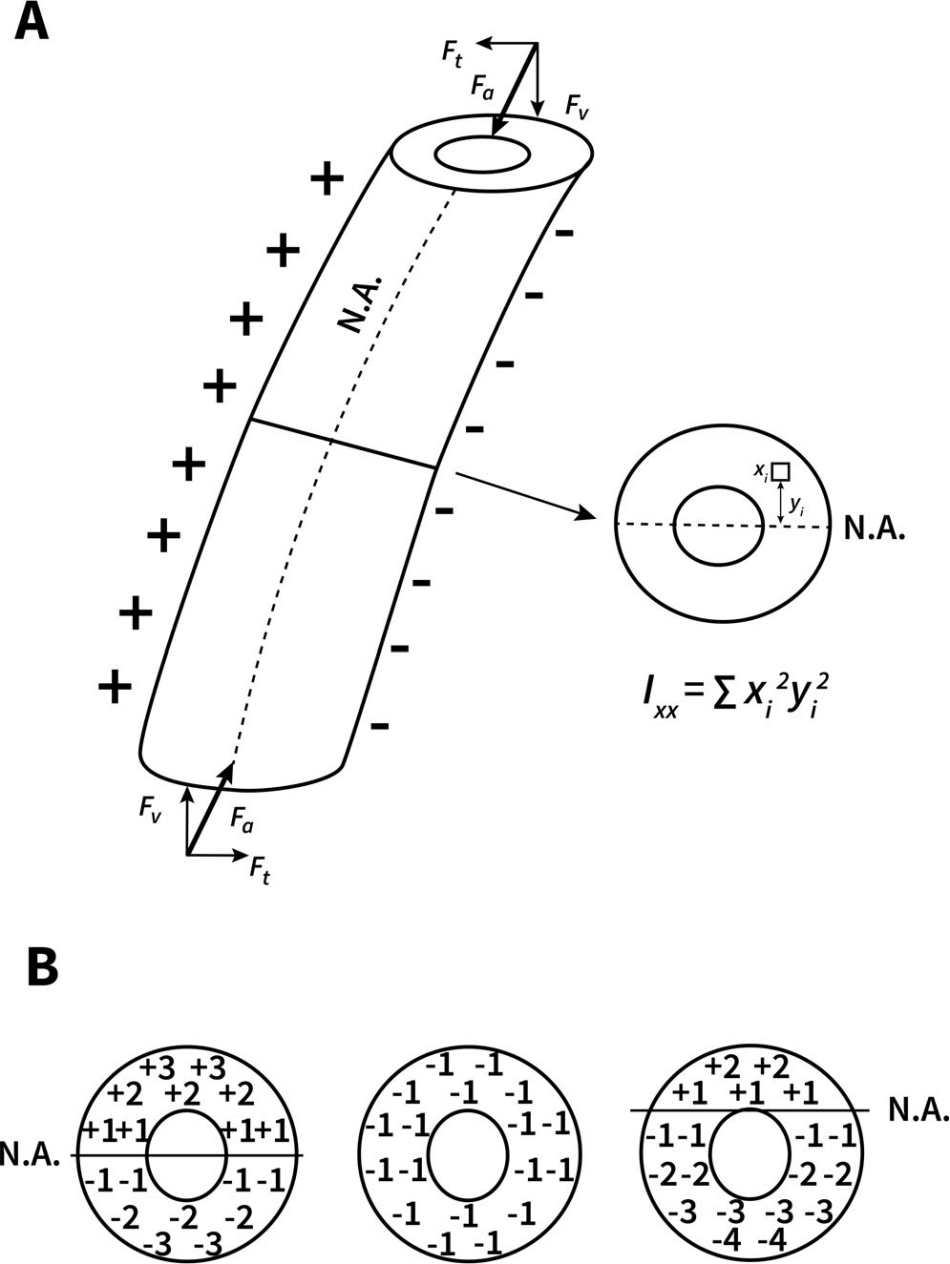
Idealized curved cylindrical beam‐like bone subject to loading at proximal end. (A) Compressive axial forces (*F*_a_) are resolved into transverse (*F*_t_) and vertical (*F*_v_) components. N.A., neutral axis around which compressive (−) and tensile (+) strains occur. Bending moment at mid‐shaft cross section is counteracted by second moment of area, *I*. The ground reaction force is shown at the lower end of the curved beam. (B) Effect of axial compression on N.A. position. On the left, a section under pure bending shows hypothetical values of compression (−) and tension (+). Superimposition of an evenly distributed compressive force of −1 shifts the N.A. towards the tension cortex (right), thus reducing strain magnitudes in that region. Redrawn from Lieberman *et al*. (2004) with permission from John Wiley & Sons, Inc.

Since the early 1970s, artiodactyl calcanei (sheep, *Ovis aries*; deer, *Odocoileus hemionus hemionus*) have become well‐established as experimental and natural models for studying adaptation, functional morphology, and evolution of functional relationships of cortical (compact) and trabecular (cancellous) bone (Lanyon, 1973, 1974; Skerry & Lanyon, 1993, 1995; Skedros *et al*., 1994a, 2006b, 2007a, 2009, 2011a, 2012b, 2019, 2023, 2024a; J. G. Skedros, J. T. Cronin, C. S. Mears & B. W. Richards, in preparation; Thomas *et al*., 1996; Skedros, Su & Bloebaum, 1997; Su *et al*., 1999; Skedros, Mason & Bloebaum, 2001; Skedros, Hunt & Bloebaum, 2004, 2005; Skedros, 2005; Skedros & Baucom, 2007; Skedros, Sorenson & Jenson, 2007b; Kim, Clement & Cunningham, 2008; Cunningham & Black, 2009; Skedros, Kiser & Mendenhall 2011c; Sinclair *et al*., 2013; Keenan, Mears & Skedros, 2017; Willie *et al*., 2020; Main, Simons & Lee, 2021; Leiss *et al*., 2024; Weiner & Shahar, 2024). Calcanei of less‐typical artiodactyls (e.g. Saiga antelope, *Saiga tartarica*) have also been used in this context (Jing *et al*., 2021, 2024; Liu *et al*., 2025). The artiodactyl calcaneus is an attractive model because: (*i*) it undergoes extensive secondary remodelling with Haversian systems (secondary osteons), similar to that seen in humans, other primates, and many larger mammals (Skedros *et al*., 2013a,b, 2014, 2023); (*ii*) it is well suited for *in vivo* strain measurements due to minimal surrounding muscles and soft tissue; (*iii*) it has relatively simple muscle and ligament attachments; and (*iv*) it is loaded like a simple lever at the hinge‐like talocrural (ankle or hock) joint, effectively resembling a short cantilevered beam (Skerry & Lanyon, 1995; Skedros *et al*., 2001, 2011d, 2019) (Figs 1 and 2). These recent studies have shown that sheep and deer calcanei have striking regional heterogeneity in their structural and material properties, which correspond to habitual strain‐mode distributions (tension, compression, and shear). Specifically, the plantar cortex typically experiences net tension, while the dorsal cortex undergoes net compression during peak loading in normal locomotion (Skedros *et al*., 2019). As a result, the shaft (tuber) region of the calcaneus displays distinct zones dominated by one of the three primary strain modes at different times during the gait cycle. Shear strain is most prevalent along the medial and lateral cortices, coinciding with the neutral axis during bending. In this context, ‘strain’ refers to mechanical deformation. Compression usually generates the highest strain magnitudes (Lieberman, Polk & Demes, 2004) (Fig. 3), as observed in the dorsal cortices of sheep and deer calcanei (Lanyon, 1974; Skedros *et al*., 2019). Among the various mechanical stimuli that a bone might experience, strain is considered the primary factor driving adaptive responses (Rubin & Lanyon, 1984b; Lanyon, 1987; Ehrlich & Lanyon, 2002). In bone, strain‐induced fluid flow dynamics that differ between compression, tension, and shear environments are likely the most proximate stimuli affecting cellular activities that mediate functional adaptations in these regions (Judex, Gross & Zernicke, 1997b; Cowin 2002; Fan *et al*., 2016; Skedros, Doutré & Weaver, 2016; van Tol *et al*., 2020).

**Fig. 4:**
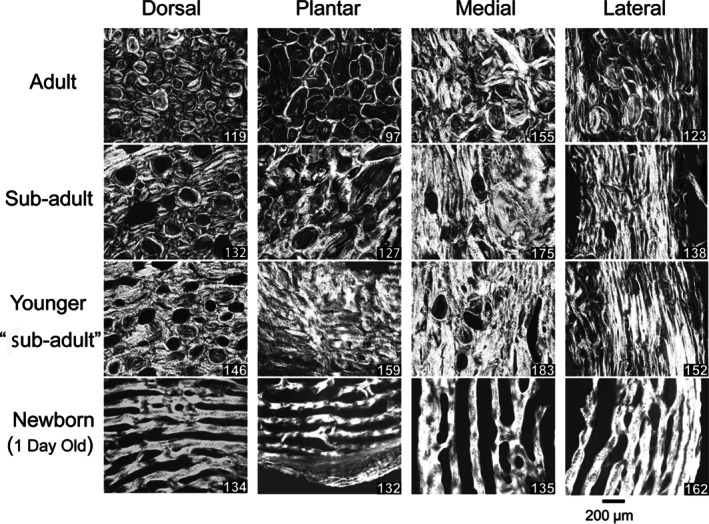
Circularly polarized images of transverse sections from a growth series of sheep calcanei. Images were taken at the 50% shaft region (Fig. 2) of sheep calcanei from an adult (top row), sub‐adult (second row), and younger animals as labelled. On the lower right of each image is the weighted‐mean grey level (WMGL), which represents predominant collagen fibre orientation (CFO) for the mineralized tissue. Reproduced from Skedros *et al*. (2007a) with permission of John G. Skedros.

**Fig. 5:**
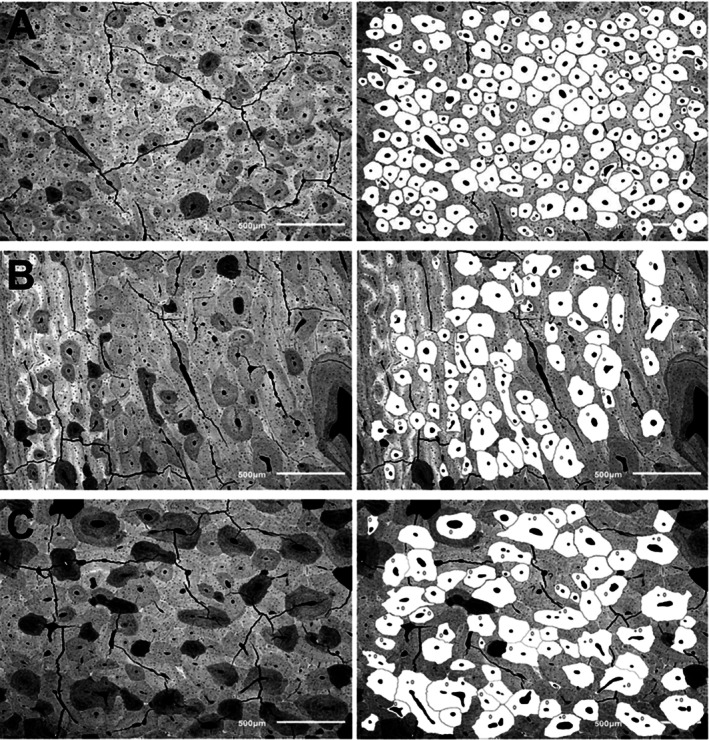
Backscattered electron images of transverse sections of dorsal cortex (A), neutral axis region (B), and plantar cortex (C) of adult deer calcaneus. The images on the right show the secondary osteons (Haversian systems) in white. Reproduced from Keenan *et al*. (2017) with permission of John G. Skedros.

Variations in material (e.g. histological) and structural characteristics are often used to interpret a bone's load history and assess its functional adaptations (Table 1). In sheep and deer calcanei, there are dramatic regional variations in tissue organization and construction that appear to be strain related. These include statistically and mechanically significant differences between their plantar ‘tension region’ and dorsal ‘compression region’ in cortical thickness, mineral content (per cent ash), microstructure (e.g. secondary osteon collagen/lamellar morphotypes and population densities; osteon size and shape), and nano/molecular structure [e.g. predominant collagen fibre orientation (CFO), orientations of mineral crystallites and collagen cross‐link densities] (Figs 4 and 5) (Gunasekaran *et al*., 1991; Skedros, Mason & Bloebaum, 1994b; Skedros *et al*., 2004, 2006a, 2007a, 2009, 2024a; Keenan *et al*., 2017). In adults, the dorsal cortex shows abundant highly birefringent secondary osteon collagen/lamellar morphotypes considered to be compression‐related adaptations, while the plantar image shows abundant darker secondary osteon morphotypes considered to be tension related (Skedros *et al*., 2009) (Fig. 4). As reported by Skedros *et al*. (2007a), the weighted‐mean grey‐level (WMGL) difference (representing CFO differences) between the dorsal (compression) and plantar (tension) regions are statistically significant (*P* < 0.01), with more oblique‐to‐transverse CFO (i.e. compression adapted) in the dorsal cortex of adult and sub‐adult deer and sheep calcanei (Fig. 4). For young sub‐adult bones, a similar trend was observed in the sheep calcaneus but was not significant in fawn deer (Skedros *et al*., 2004). As expected, grey level/CFO comparisons between the medial and lateral cortices (i.e. neutral axis region) were not statistically significant in either group (*P* > 0.15).

**Table 1:** Biomechanically important structural and material characteristics in diaphyseal bone hierarchical organization [excluding trabecular (cancellous) bone].

**1. Structural characteristics**
Bone length
Diaphyseal curvature
Cross‐sectional shape and robusticity [e.g. moments and axes of area (inertia), relative cortical area]
Average and regional cortical thickness variations
**2. Material characteristics**
Mineral content (% ash)
*Microstructure*
Secondary osteon population density and fractional area (On.N/T.Ar, On.Ar/T.Ar)
Secondary osteon cross‐sectional area, shape, and orientation
Secondary osteon morphotypes (e.g. bright, alternating, parallel‐fibred, hooped)
Mineral heterogeneity (e.g. relatively highly mineralized interstitial bone, young osteons, etc.)
Collagen fibre orientation (CFO) heterogeneity
Porosity (e.g. Haversian canals, primary vascular canals)
Lamellar organization of various osteon morphotypes
Variations in primary histological organization (e.g. laminar *versus* reticular vascular patterns in fibrolamellar/plexiform bone, distribution of circumferential lamellar bone)
Osteocyte population density, osteocyte lacuna‐canalicular geometries
*Nanostructure*
Predominant collagen fibre orientation (CFO), collagen density
Types and densities of collagen molecular cross‐links
Mineral crystallite and ‘tesselle’ orientation, size, and heterogeneity
Spatial distribution of non‐collagenous proteins (e.g. osteopontin and osteocalcin)

**Fig. 6:**
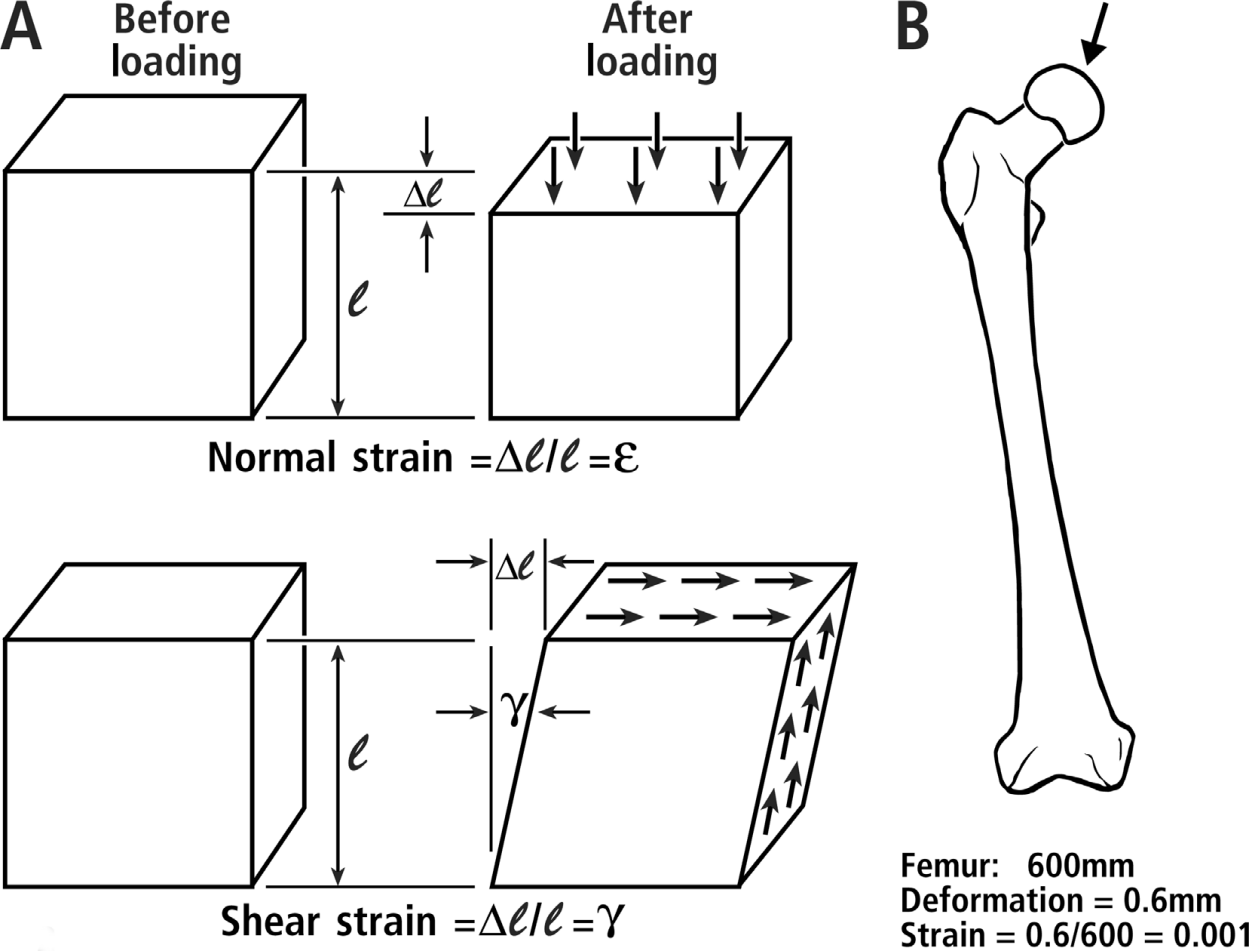
Definition of strain deformation as the change in length (∆*l*) divided by the original length (*l*). (A) Strain is shown in compression stress (top), and shear stress (bottom), where true shear strain is measured as a change in angle γ (in radians). Bone adaptation studies typically use ‘engineering shear strain’ defined as the tangent of γ and calculated as the maximum length of deformation divided by the perpendicular length in the plane of force application, which usually makes it easier to calculate (Skedros *et al*., 2019). (B) Human femur strained at 1000 microstrain (0.001), which is 0.1% deformation.

The earliest use of strain gauges placed directly on the surface of a limb bone to quantify deformations (strains) can be traced to the unpublished work of F. Gaynor Evans and colleagues (Evans, 1953; Szivek & Gharpuray, 1999). In these studies, a strain gauge was attached to the tibia of a live dog to record bone strains during natural gait. This was a revolutionary advance in the field of bone biomechanics, surpassing earlier indirect methods for estimating bone deformation (Bouvier, 1985; Hoshaw *et al*., 1997; Al Nazer *et al*., 2012). Mechanical strain, whether in compression or tension, is defined as the change in length of a loaded structure, expressed as a percentage of its initial (unloaded) length (Fig. 6). In isotropic materials loaded axially, strain is proportional to stress (force per unit area), according to Hooke's law.

Lanyon & Smith (1969, 1970) advanced the work of F. G. Evans by developing and employing strain gauges *in vivo*, attaching them directly to the surface of tibial diaphyses of live sheep during walking. Lanyon (1971, 1972) subsequently reported *in vivo* strain data from gauges on lumbar and thoracic vertebral bodies of sheep during locomotion. These seminal studies provided the foundation for the introduction of the sheep calcaneus as a model for studying bone adaptation (Lanyon, 1973, 1974). Lanyon noted that the sheep tibia and vertebral bodies had load‐induced complexities, making it difficult to draw clear conclusions about how local strains might influence bone adaptations. Therefore, he identified a more simply loaded bone better suited for *in vivo* strain gauge application, the sheep calcaneus, which led to a series of influential studies using this model (Lanyon, 1973, 1974; Skerry & Lanyon, 1993, 1995; Thomas *et al*., 1996). This work formed the foundation of important contributions to bone research, leading to the development and exploration of other experimental/natural models, including the sheep radius, sheep tibia, turkey ulna, and rat ulna (Lanyon, Goodship & Baggot, 1976; Lanyon & Bourn, 1979; Lanyon, Magee & Baggot, 1979; O'Connor, Lanyon & MacFie, 1982; Lanyon & Rubin, 1984; Rubin & Lanyon, 1984b; Torrance *et al*., 1994; Mosley & Lanyon, 1998; Ehrlich *et al*., 2002). Notably, these and other *in vivo* strain studies on long bones contributed to overturning Harold Frost's ‘Flexion Neutralization Theory’, which was influential during the 1960s and 1970s. Frost proposed that limb bones naturally develop to minimize bending and favour compression loading, effectively neutralizing tension strains to reduce fracture risk (Frost, 1963, 1983; Bouvier, 1985; Betram & Biewener, 1988; Martin & Burr, 1989). That hypothesis was ultimately rejected by *in vivo* studies showing that the limb bones of most terrestrial vertebrates experience non‐uniform strain distributions, often dominated by bending (Currey, 1984; Betram & Biewener, 1988; Fritton & Rubin, 2001; Currey, 2002).

The regional structural and material variations in sheep and deer calcanei (Table 2) are believed to represent the outcome of strong natural selection for avoiding fracture by accommodating marked regional differences in mechanical requirements. These regional variations are associated with habitual differences in strain modes, magnitudes, and other strain characteristics of the differing load histories of the main cortical regions of these bones due to their habitual unidirectional bending (Skedros *et al*., 2006a, 2019, 2024a).

**Table 2:** Structural and material differences between dorsal, plantar, and medial‐lateral (‘neutral axis’) cortices of deer and sheep calcanei. For trabecular bone data, see Sinclair *et al.* (2013) and Skedros *et al.* (2012b). D = dorsal, P = plantar, M = medial, L = lateral; NA = data not available; CFO, collagen fibre orientation; OPD = secondary osteon population density [all osteon data are based on secondary osteons (Haversian systems) only]. Grey highlight indicates statistically significant differences. ‡ Indicates when the comparison is not the expected adaptation for ambient strain mode (where the expected pattern is compression in D, tension in P; and M and L should experience approximately equivalent strain). † *Ex vivo* strain data in deer calcanei show that the neutral axis (M and L cortices) is somewhat oblique (see Fig. 1A) (Skedros *et al.*, 2019), which places the medial cortex in some compression and the lateral cortex in some tension, potentially explaining these M *versus* L differences. ^+^ New remodelling events refer to resorption cavities created by osteoclasts during the osteon remodelling process and forming osteons (Skedros *et al.*, 2001). Data sources: Gunasekaran *et al.* (1991); Skedros *et al.* (1994a,b, 1997, 2001, 2004, 2005, 2007a); Skedros (2005); Keenan *et al.* (2017).

Characteristic	Deer calcaneus		Sheep calcaneus	
	Dorsal *vs*. plantar	Medial *vs*. lateral	Dorsal *vs*. plantar	Medial *vs*. lateral
Cortical thickness	D > P	M = L	D > P	M = L
Predominant CFO (>oblique‐to‐transverse)	D > P	M = L	D > P	M = L
Mineral content	D > P	M = L	D > P	M = L
New remodelling events ^+^	P > D	M = L	P > D	M = L
Porosity	P > D	M = L	P > D	M > L
OPD	D > P	M > L ^‡ †^	D > P	M > L ^‡ †^
Osteon size	P > D	L > M ^‡ †^	P > D	L > M ^‡ †^
Non‐circular osteons	P > D	M = L	P = D	M = L
Osteocyte lacuna density	D > P	M = L	D > P	M = L
Collagen cross‐links	D > P	NA	NA	NA

Elucidating the mechanisms governing the emergence of the highly heterogeneous regional variations in structural and material characteristics of artiodactyl calcanei is important because: (*i*) these characteristics are considered extreme manifestations of the natural adaptive capacity of bone tissue that is present, but often less marked, in limb bones of many mammalian and avian species, and (*ii*) deficiencies in this adaptive capacity contribute to skeletal fragility, and thus are of clinical relevance due to the exponential increase in frequency of fragility fractures with age (Biewener, 1993; Melton, 1996; Duan *et al*., 2003; Diab *et al*., 2005; Mayhew *et al*., 2005; von Kroge *et al*., 2022).

Therefore, it is important to use models like the cantilevered‐bending artiodactyl calcaneus to attain a greater understanding of how the mechanobiology of a bone is optimized by the coupling of structural and material characteristics (Skedros *et al*., 2023). The artiodactyl calcaneus: (*i*) is habitually loaded in bending during normal locomotor loads, a common loading condition in limb bones of many species (Rubin & Lanyon, 1984a; Biewener, Swartz & Bertram, 1986; Biewener & Bertram, 1993a; Demes *et al*., 1998, 2001; Lovejoy *et al*., 2002; Lieberman *et al*., 2003; Moreno, Main & Biewener, 2008; Milne, 2016), and (*ii*) resembles the cantilevered beam‐like loading models that depict other bones and bone regions, including the hominid proximal femur (Lovejoy, 1988; Rafferty, 1998; Lovejoy *et al*., 2002; Mayhew *et al*., 2005; Skedros & Baucom, 2007; Ruff & Higgins, 2013; Sinclair *et al*., 2013; Nawathe *et al*., 2015; Kersh *et al*., 2018; Skedros *et al*., 2023) and potoroo (*Potorous tridactylus*) calcaneus (Biewener *et al*., 1996). The artiodactyl calcaneus model has been used to advance understanding of the strengths and limitations of the normal functional morphology of the human proximal femur as a quasi‐cantilevered beam and how age‐related changes in its strain environment increase its susceptibility to low‐energy fractures (Bell *et al*., 1999; Mayhew *et al*., 2005; Sinclair *et al*., 2013; Skedros *et al*., 2023).

Studies of appendicular bones that are simply loaded in bending can help elucidate both the mechanical advantages and lesser‐known physiological benefits of bending. For example, directional control of bending provides load predictability, ensuring a consistent strain milieu that promotes reliable nutrient delivery to bone cells *via* strain‐induced interstitial fluid flow. Hence, in both physiological and mechanical contexts, predictable loading is more favourable than unpredictable loading (Betram & Biewener, 1988; Skedros *et al*., 1996; Judex *et al*., 1997b; Srinivasan & Gross, 2000; Willie *et al*., 2020). However, bending also introduces potentially deleterious strain distribution that must be accommodated by structural and material modifications. These accommodations are necessary because bending produces regional concentrations of dominant shear and tension strains, which tend to cause more microdamage than compression (Boyce *et al*., 1998; Reilly & Currey, 1999; Lambers *et al*., 2014; Tang *et al*., 2015). This may explain the non‐uniform structural and material organization of the artiodactyl calcaneus (Table 2) (Donnelly *et al*., 2012; Skedros *et al*., 2014; Zimmermann *et al*., 2014; Allahyari *et al*., 2023).

Specific strain modes or related stimuli, including strain gradients, streaming potentials, and directional fluid flow, are linked to the most proximate signals that regulate morphological bone adaptation (Judex *et al*., 1997a; Srinivasan & Gross, 2000; Skedros *et al*., 2016; van Tol *et al*., 2020). These stimuli can activate distinct cellular responses, leading to differential bone remodelling/modelling responses (Yeh & Rodan, 1984; Skerry *et al*., 1990; Ingram *et al*., 1994; Rubin *et al*., 1996b; Su, Borke & Donahue, 1997; Terai *et al*., 1999; Ikegame *et al*., 2001). Distinguishing between tension, compression, and shear is also fundamentally important in bone formation, growth, maintenance, and senescence due to their different osteogenic capacities (Rubin *et al*., 1996b; Mayhew *et al*., 2005; Hart *et al*., 2017; Manandhar *et al*., 2023; Rooney *et al*., 2023). Experimental evidence is clear – bone cells can perceive and respond to differences in strain mode (Zhong *et al*., 2013; Li *et al*., 2015; Kanzaki *et al*., 2019; Ei Hsu Hlaing *et al*., 2020). Studies that overlook this possibility likely failed to detect evidence of it due to insufficient examination of bone material organization, cellular activity, and biochemical expression (Morimoto, Ponce de Leon & Zollikofer, 2011; Lad, McGraw & Daegling, 2019; Javaheri *et al*., 2020; Baleani *et al*., 2024).

Many studies referencing artiodactyl calcanei as biomechanical or experimental models have done so under the assumption that these bones function like simply loaded, short‐cantilevered beams with predominantly unidirectional loading. Given the close morphological similarities of calcanei and talocrural mechanics observed across a variety of terrestrial artiodactyls (Schmid, 1972; Prummel & Frisch, 1986; France, 2009; Fisher, Scott & Adrian, 2010), this simplified load environment likely applies across a broad range of body sizes, from the smallest (mouse deer, *Tragulus javanicus*) to the largest (giraffe, *Giraffa camelopardalis*). However, there is limited support for the view that this strain distribution is habitual (i.e. spatially and temporally stereotypical). *In vivo* strain data exist only from sheep calcanei studies (Lanyon, 1973, 1974) and more recent *ex vivo* data from mule deer (*Odocoileus hemionus hemionus*) (Su *et al*., 1999; Skedros *et al*., 2019, 2023). The strain data reported in these studies are limited by being obtained from only one or a few strain gauges per bone, with measurements only during walking or trotting. As shown by Gross, McLeod & Rubin (1992), a minimum of three triple‐rosette strain gauges placed at approximately equidistant locations are needed to create a valid finite element model for predicting strains at other locations on and within the cortex of the same transverse section of a limb bone. Only two studies have approached this level of analysis, using five rosettes on the mid‐diaphysis of mule deer calcanei in an *ex vivo* model (Su *et al*., 1999; Skedros *et al*., 2019). However, to date, the only finite element model of an artiodactyl calcaneus is that of the saiga antelope (*Saiga tatarica*) (Liu *et al*., 2025). In that model, dorsal *versus* plantar differences in the stress/strain environment were not considered and no strain gauge measurements were attempted on actual bones either *in vivo* or *ex vivo*. Consequently, in view of these limitations, the long‐held view of sheep and deer calcanei as habitually simply loaded ‘tension/compression bones’ remains under‐supported and controversial.

A major and often‐cited challenge to the ‘tension/compression bone’ model (plantar/dorsal cortices) comes from McMahon, Boyde & Bromage (1995), who used *in vivo* strain data from Lanyon (1973) and their own qualitative histological observations, which showed the absence of expected regional patterns of predominant CFO in circularly polarized light (CPL) images of thin transverse sections of calcanei of domesticated sheep. The ‘expected’ differences would have been oblique‐to‐transverse CFO (i.e. brighter/lighter grey levels in CPL) in the dorsal ‘compression region’ *versus* more longitudinal CFO (i.e. darker in CPL) in the plantar ‘tension region’ (Portigliatti Barbos, Bianco & Ascenzi, 1983; Carando *et al*., 1989; Boyde & Riggs, 1990; Riggs, Lanyon & Boyde, 1993) (Fig. 4). Unexpectedly, McMahon *et al*. (1995) observed generally more longitudinal CFO (presumably ‘tension adapted’) in both dorsal‐lateral and plantar‐lateral cortices, casting doubt (discussed below) on the simple beam‐bending model. Their findings were prominently cited by Currey (2002) in his authoritative textbook, where he echoed their concerns about the suitability of the sheep calcaneus as a model for studying bone adaptation under habitual loading. Other investigators have also referenced McMahon *et al*. (1995), arguing that the artiodactyl calcaneus model and regional variations in predominant CFO can be misleading when inferring habitual loading, especially when distinguishing bending from torsion (Boyde & Jones, 1998; Boskey, Wright & Blank, 1999; Kalmey & Lovejoy, 2002; Bromage *et al*., 2003; de Margerie *et al*., 2005; Main, 2007; Ramasamy & Akkus, 2007; Rothschild & Panza, 2007; McFarlin *et al*., 2008; Warshaw *et al*., 2017; Main *et al*., 2021). By contrast, their study has been misinterpreted as supporting expected CFO adaptations to tension and compression (Stockhausen *et al*., 2021). Therefore, McMahon *et al*. (1995) is influential not only because it challenges decades of work showing regional CFO and structural adaptations in the sheep calcaneus consistent with unidirectional bending, but also because it questions the broader validity of using the artiodactyl calcaneus as a relatively simple model for studying bone adaptation.

While completing our first *ex vivo* study on the strain environment of adult mule deer calcanei (Su *et al*., 1999), we identified errors in the strain data reported in Table 4 of McMahon *et al*. (1995), which were extracted from the strain plots in fig. 4 of Lanyon (1973). These data are reproduced in Table 3A and corrected in Table 3B and detailed in Section II.

**Table 3:** (A) Strain data (με) reported by McMahon *et al.* (1995) from Lanyon's (1973) animals S34 and S35. (B) Corrected data. McMahon *et al.* (1995) misreported both the strain gauge locations and the signs (− or +) of the strain values. In the corrected data (B), the negative (−) values accurately represent compression microstrain, and positive (+) values represent tension microstrain. Strain values are from continuous strain recordings during walking and trotting shown in fig. 4 in Lanyon (1973). Data in (A) are reproduced from McMahon *et al.* (1995) with permission from John Wiley and Sons, Inc. and from Lanyon (1973) with permission from Elsevier Inc.

(A) Data reported in McMahon *et al*. (1995)				
Time (s)	Specimen S34		Specimen S35	
	Plantar‐lateral (gauge 1)	Dorsal‐lateral (gauge 2)	Plantar‐lateral (gauge 1)	Dorsal‐lateral (gauge 2)
0.0	0.00	0.00	0.00	0.00
0.1	+83.50	−43.60	+41.50	−74.70
0.2	+225.45	−50.10	+190.90	−16.60
0.3	+117.00	−25.10	+24.90	+20.80
0.4	−10.00	+58.50	−6.70	+43.20
0.5	0.00	0.00	0.00	0.00

(B) Corrected data (present review)				
Time (s)	Specimen S34		Specimen S35	
	Plantar‐lateral (gauge 1)	Dorsal‐lateral (gauge 2)	Plantar‐lateral (gauge 1)	Dorsal‐lateral (gauge 2)
0.0	0.00	0.00	0.00	0.00
0.1	+43.60	−83.50	+74.70	−41.50
0.2	+50.10	−225.45	+16.60	−190.90
0.3	+25.10	−117.00	−20.80	−24.90
0.4	−58.50	+10.00	−43.20	+6.70
0.5	0.00	0.00	0.00	0.00

## II. THE CONCLUSIONS OF McMAHON *et al.* (1995) AND RE‐ANALYSIS OF LANYON'S (1973) DATA

### (1) Re‐analysis of the strain data used by McMahon *et al.* (1995)

**Fig. 7:**
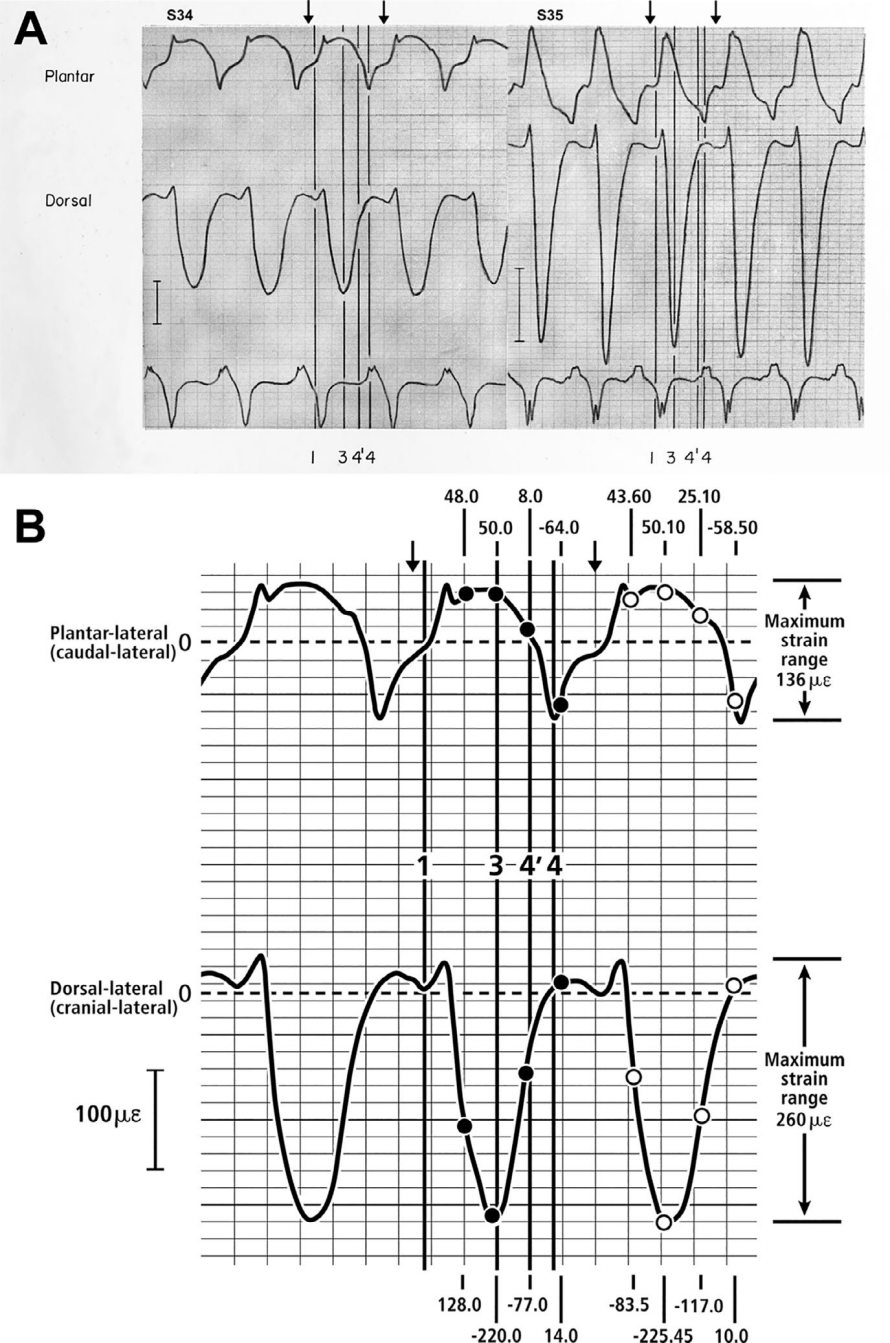
Photograph (A) of portion of original Fig. 4 from Lanyon (1973) and drawing (B) based on these curves. (A) Digitally enhanced image of the original strain plots for animals S34 (left) and S35 (right). The numbers 1, 3, 4′, and 4 are original designations from Lanyon (1973). Arrows added to the top of each plot indicate one gait cycle used for analysis in Table 4. These arrows intentionally extend beyond time points 1 and 4, which do not include a complete swing phase. (B) Re‐drawing of a portion of strain plots from animal S34, showing three gait cycles (stance/swing cycles). The four dark vertical lines denote Lanyon's (1973) original time points: (1) swing phase (no weight bearing), (3) mid‐stance (full weight bearing), (4′) when initial limb lift‐off was observed near the conclusion of stance phase, and (4) limb lift‐off. Note that the duration from 1 to 4 is not a complete gait cycle; the arrows added at the top of this figure represent one complete gait cycle analysed in Table 4. Dots in (B) show likely points where McMahon *et al*. (1995) extracted data, which are reproduced here in Table 3A. The time courses for the shaded areas added for the plantar‐lateral gauges in Fig. 8 are consistent with Lanyon's interpretations. The accurate numerical values that are added on each of the two curve pairs at the far‐right side (white dots) are at locations that do not fall on the vertical lines of the grid but fall within 5% of McMahon *et al*.'s (1995) reported values (Table 3). Black dots indicate the accurate numerical values for comparison. Redrawn strain plots are from Lanyon (1973) with permission from Elsevier, Inc. for reproduction of the original.

McMahon *et al*. (1995) primarily referred to strain data from animals S34 and S35 in Lanyon (1973), which were obtained from two single‐element strain gauges on each bone during walking (Lanyon, 1973). McMahon *et al*. (1995) obtained strain data directly from Lanyon's (1973) representative graphs of *in vivo* strain data from animals S34 and S35 (the only animals with both dorsal and plantar gauges) and I repeated this process. The data extracted are from points where the strain profiles (i.e. the curvilinear strain plots) intersected the vertical lines seen in Lanyon's Fig. 4 and at 0.1 s intervals shown on that figure (Fig. 7A). I analysed a complete gait cycle to reduce any risk of bias. The strain gauges used for this analysis were referred to by McMahon *et al*. (1995) as the ‘cranio‐lateral’ and ‘caudo‐lateral’ gauges, which are designated here in preferred anatomical terminology as the dorsal‐lateral (or cranial‐lateral) and plantar‐lateral (or caudal‐lateral) gauges, respectively.

**Fig. 8:**
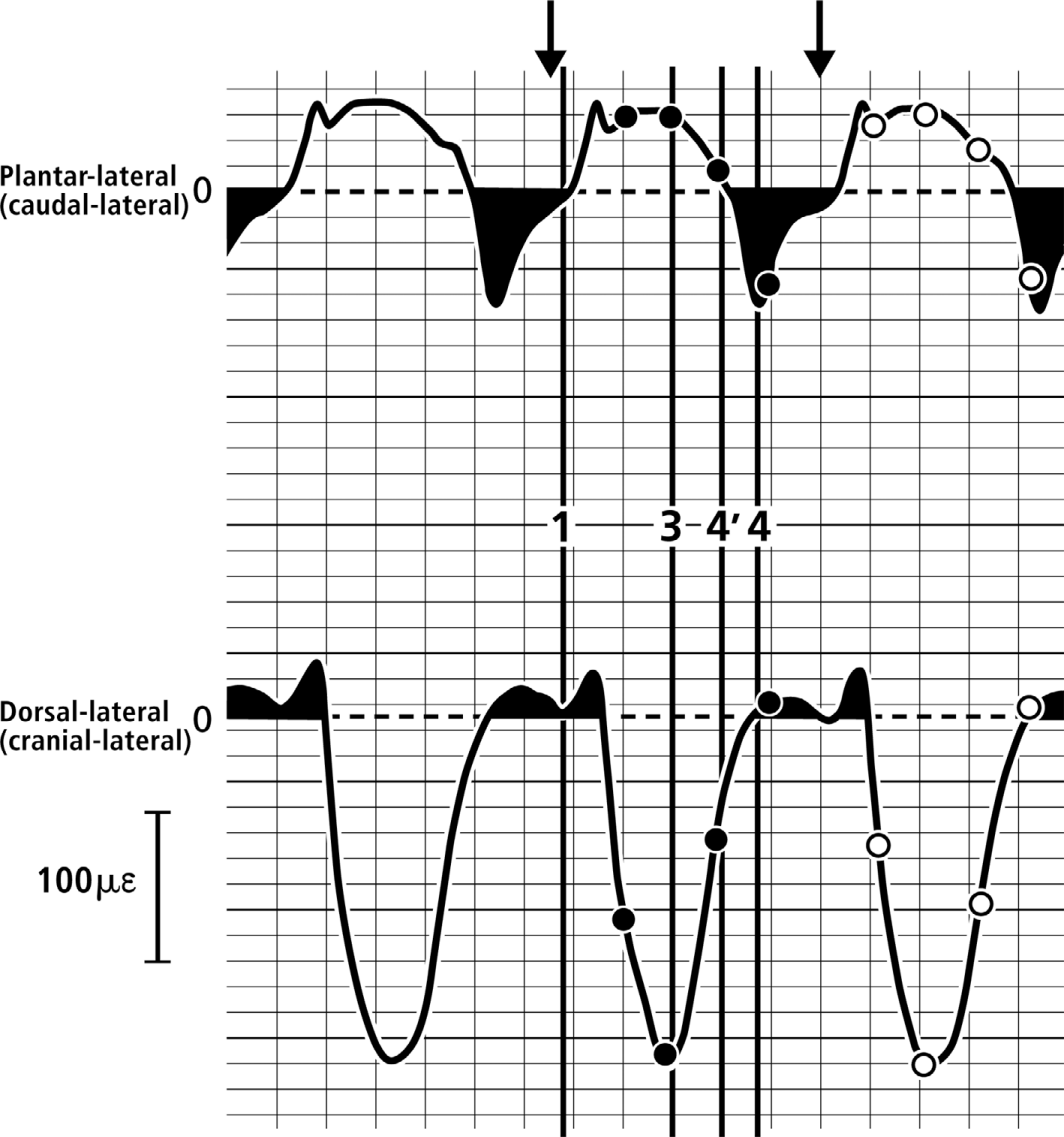
Re‐drawn strain curves recorded at two gauges on the calcaneus of animal S34 from Lanyon (1973). The shaded areas show the strain‐mode reversal phases for each gauge location, specifically, values below the zero‐strain line (dashed line) at the plantar‐lateral gauge and above the zero‐strain line at the dorsal‐lateral gauge. The non‐reversal and reversal phases represent the weight‐bearing (stance) and non‐weight‐bearing (swing) phases, respectively. The two arrows at the top indicate one gait cycle that was used for the analysis in Table 4. As shown in Fig. 7, these arrows intentionally extend beyond Lanyon's 1 and 4 time points, as this interval does not encompass a complete swing phase. The complete gait cycle analysed here helps reduce bias that would favour the compression/tension dorsal/plantar concept of habitual loading.

The below analysis focuses on animal S34, but similar issues are also present for animal S35 (Table 4 in McMahon *et al*., 1995). For animal S34, I redrew the strain profiles for three of the five loading cycles in Lanyon's (1973) fig. 4 (Fig. 7B herein) to enable digitization and closer examination at higher magnification. Although Lanyon (1973) did not indicate the zero strain locations with clear transverse lines in the plots of his fig. 4, they could be closely approximated by referring to the late swing phase of gait that he denoted as ‘1’ of the four gait instants in this figure and are shown as vertical lines in Figs 7 and 8 herein. Notably, the strain plots from the dorsal‐lateral cortices of animals S34 and S35 have similar shapes, allowing increased confidence in identifying the zero‐strain locations. These locations correspond with those shown in Fig. 2 of McMahon *et al*. (1995), which is their re‐drawing of strain plots from Lanyon's animal S35.

**Table 4:** Strain durations, overall predominance, and peak strain in compression and tension from one gait cycle for animals S34 and S35 from Lanyon (1973). The strain data for one representative gait cycle (indicated by arrows above the strain curves in Figs 7 and 8) are shown for each of the dorsal and plantar gauges. Data extracted from fig. 4 in Lanyon (1973) reproduced in Fig. 7A herein. C = ‘compression cortex’, T = ‘tension cortex’. *, the relative area under the curves in one gait cycle (shaded in Fig. 8) is provided here as an estimate of the overall predominance of compression or tension. The area under a curve represents an estimate of strain magnitude and duration. Y = consistent with the net compression/tension simplification for the dorsal cortex, or net tension/compression simplification for the plantar cortex. N = not consistent with the net compression/tension simplification for the dorsal cortex, or net tension/compression simplification for the plantar cortex.

	% of gait cycle in:		% area under curve* in:		Peak strain (με) in:		Peak strain ratio	% gait‐time‐adjusted peak strain ratio	Area‐under‐curve‐adjusted peak strain ratio
	Compression	Tension	Compression	Tension	Compression	Tension			
Animal S34									
Dorsal‐lateral (C)	52%	48%	92%	8%	220	32	C/T = 6.9^Y^	C/T = 7.4^Y^	C/T = 79.1^Y^
							[220/32]	[(220 × 0.52)/(32 × 0.48)]	[(220 × 0.92)/(32 × 0.08)]
Plantar‐lateral (T)	35%	65%	30%	70%	787	50	**T/C = 0.6** ^**N**^	T/C = 1.2^Y^	T/C = 1.5^Y^
							**[50/78]**	[(50 × 0.65)/(78 × 0.35)]	[(50 × 0.7)/(78 × 0.3)]
Animal S35									
Dorsal‐lateral (C)	53%	47%	94%	6%	267	26	C/T = 10.2^Y^	C/T = 11.6^Y^	C/T = 160.9^Y^
							[267/26]	[(267 × 0.53)/(26 × 0.47)]	[(267 × 0.94)/(26 × 0.06)]
Plantar‐lateral (T)	48%	51%	38%	62%	45	73	T/C = 1.6^Y^	T/C = 1.7^Y^	T/C = 2.6^Y^
							[73/45]	[(73 × 0.51)/(45 × 0.48)]	[(73 × 0.62)/(45 × 0.38)]

Using a magnifying lens and digital calliper, the original strain plots were also measured directly from the page of the journal wherein they appeared by myself and two colleagues who were blinded to the hypothesis and used the same methods. Inter‐observer analysis revealed that our direct calliper measurements from the published images were within 2% of the values obtained using *ImageJ* on the digitized strain plots. Two additional colleagues independently reviewed and confirmed the accuracy of our results. It appears that McMahon *et al*. (1995) made errors in assigning the correct strain mode (positive for tension, negative for compression) and mismatched strain magnitudes with gauge location in Table 4 of their study (Table 3A). We obtained absolute strain values from the original plots (Lanyon, 1973) for animals S34 and S35 that were within 5% of the absolute strain values reported in Table 4 of McMahon *et al*. (1995), despite their misassignments.

The two versions of strain data from animals S34 and S35 (Table 3) reveal and correct errors in the data used by McMahon *et al*. (1995), including inaccuracies in strain magnitudes, sign conventions (+/−), and gauge locations. By convention, compression strains are negative and tension strains are positive (Fig. 3). Figure 7B provides one example of my re‐analysis. Unmistakable evidence of errors in the tabulated data from McMahon *et al*. (1995) can be seen: some of their absolute strain values for animal S34 (Table 3A) exceed the strain ranges (right side of Fig. 7B) shown in Lanyon's strain plots. For example, for the plantar‐lateral gauge of animal S34, McMahon *et al*. (1995) report a tensile strain of 225.45 με when the maximum strain range at this gauge did not exceed 140 με. Additionally, the dorsal‐lateral (compression region) stance‐phase absolute strain values for S34 reported by McMahon *et al*. (1995) do not exceed 51 με, which is unexpectedly low. In this region, compression strains during the weight‐bearing phase of gait typically exceed 150 με.

Figure 8 highlights portions of the strain plots from animal S34 (Fig. 7) where strain mode reversals occur, reflecting the weight‐bearing to non‐weight‐bearing transition. These reversal phases mark the point where plantar strains switch from tension to compression (positive to negative), and dorsal strains switch from compression to tension (negative to positive). During walking, these reversal periods account for approximately 38–44% of the full swing‐stance strain cycle at both plantar‐lateral and dorsal‐lateral gauges, indicating that the reversal (non‐weight‐bearing) phase is shorter than the non‐reversal (weight‐bearing) phase of a full cycle of walking. Hence, most of the strains at the locations analysed in Figs 7 and 8 occur during the weight‐bearing (non‐reversal) phase (i.e. 56–62% of the full non‐reversal/reversal cycle). A relatively longer duration of the weight‐bearing phase has been reported in other breeds of domesticated sheep during walking (Kim & Breur, 2008; Valentin *et al*., 2014; Costa *et al*., 2018).

At the plantar‐lateral gauge, the absolute magnitudes of the peak strains before and during the reversal phase are comparable (about +58 με and − 66 με, respectively). However, at the dorsal‐lateral gauge, the difference is dramatic: approximately −225.5 με before the reversal phase and + 35 με during the reversal phase. This asymmetry is consistent with the expectation that during weight‐bearing, locations farther from the neutral axis will experience higher strains (Fig. 3). An important consideration is the amount of time that each cortex is in net tension or net compression. As shown in Table 4, as based on data from Lanyon (1973) (Figs 7 and 8), the percentage of the entire gait cycle that the plantar‐lateral (‘tension region’) gauge is in net tension strain is 65% for animal S34, and 51% for animal S35. These results show that the ratio of peak strain indicated net compression in one of the two animals in the plantar‐lateral (‘tension’) cortex (tension/compression ratio = 0.6) (Table 4). All other data expressions had tension/compression ratios >1.0 that support the simplified net tension/compression plantar/dorsal concept for the habitual loading of the sheep calcaneus.

The data analysed from animals S34 and S35 were from strain gauges positioned on both the dorsal‐lateral (compression) and plantar‐lateral (tension) cortices, allowing direct comparison. Unfortunately, these gauges were single‐element types. This is an important limitation given that under natural conditions beam‐like bones are loaded in combinations of transverse and quasi‐axial loads, which cause strain magnitudes and directions to deviate from those seen in simple beam loading (Young, 1989; McMahon *et al*., 1995; Brassey *et al*., 2013). In artiodactyl calcanei, deviations in principal strain directions progressively increase in the vicinity of the neutral axis and from the mid‐third of the beam (where histomorphological observations are often made) to the distal and proximal ends of the beam, likely reflecting increased complexity of loading in these locations (Skedros *et al*., 1994a; Su *et al*., 1999; Skedros & Baucom, 2007; Sinclair *et al*., 2013).

Because Lanyon's (1973) single‐element plantar‐lateral (‘tension region’) gauges unexpectedly showed net compression during mid‐stance in animal S35 (although not in S34), he suggested that the expected net tension near the plantar cortex caused by the pull of the Achilles tendon (common calcaneal tendon in Fig. 1A) was neutralized by the recoil of the plantar ligament (PL). Consequently, when considering the data from the one of the two animals with single‐element plantar‐lateral gauges, Lanyon (1973) and later McMahon *et al*. (1995) rejected the view that the sheep calcaneus approximates a simply loaded cantilevered beam. By contrast, Lanyon (1974) used rosette gauges on varying lateral locations of eight calcanei, with gauges positioned closer to the plantar cortex in two bones. These rosette gauges allowed a more complete assessment of the strains on the plantar‐lateral cortex, which were clearly in net tension at mid‐stance, consistent with the ‘tension/compression bone’ concept. In fact, Lanyon (1974) seemed confident that this strain environment was habitual, leading him to support the use of the sheep calcaneus as a model for investigating the ‘trajectorial theory of trabecular bone structure’. This theory proposes that opposing tension and compression stress trajectories resemble the arched trabecular patterns that become confluent with the respective plantar and dorsal cortices (Biewener *et al*., 1996; Skedros & Baucom, 2007; Skedros *et al*., 2023) (Fig. 1B and Section IV). Notably, McMahon *et al*. (1995) did not reference Lanyon (1974).

In *ex vivo* strain gauge studies of deer calcanei (Skedros *et al*., 2019; Skedros *et al*., 2023), we found no evidence for neutralization of the net tension strains on the plantar cortex. Although we did not simulate the swing phase of gait, thus precluding detection of PL recoil effects, we did perform sequential cutting of the PL and superficial digital flexor tendon (SDFT) during weight‐bearing to investigate their load‐sharing and load‐modifying roles. Strains were measured using rosette gauges at dorsal and plantar sites during these various loading regimes. Notably, a gauge was also placed on the plantar cortex (i.e. beneath the PL) after separation (with a scalpel) of the PL from that region. The strain data summarized in Table 5 (see also Section VI.2 for safety factors in the dorsal and plantar cortices) showed that plantar strains remained in net tension throughout the entire loading cycle, even as the load was removed, contrasting with Lanyon's (1973) observations. However, his strain gauges were next to the PL, not beneath it. After separating the PL from the bone, we observed significant increases in plantar tension strains and slight decreases in dorsal‐lateral compression strains, indicating that the PL and SDFT help reduce (or modulate) strain but do not eliminate bending. These findings suggest that both of these soft tissue structures can reduce strain magnitudes, especially in the plantar cortex, rather than neutralizing bending of the calcaneal shaft.

**Table 5:** *Ex vivo* strain data from adult deer calcanei with 50 kg load (walking), and 100 kg load (running). From Skedros *et al.* (2023).

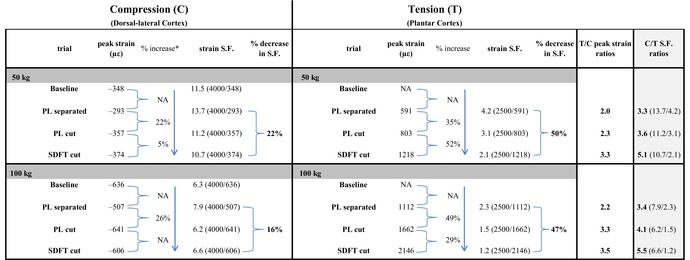

S.F. = safety factor; PL = plantar ligament; SDFT = superficial digital flexor tendon; C = compression; T = tension. * % increases in compression data are calculated using absolute values (by convention, compression strains have negative values). % increase from baseline to SDFT cut = [((larger number) – (smaller number))/(smaller number) × 100]. NA = not applicable (i.e. there is no % increase in compression/dorsal net strain); not available for plantar cortex data with intact PL (‘baseline’).

Table 6 demonstrates that the *in vivo* strain data from Lanyon (1973, 1974) in adult sheep calcanei are similar to the *ex vivo* strain data in adult mule deer calcanei reported by our group (Su *et al*., 1999; Skedros *et al*., 2019). Our *ex vivo* strain data also align with *in vivo* data reported by Skerry & Lanyon (1995) for sheep calcanei, measuring principal compressive strains during treadmill exercise, which increased hoof reaction forces to beyond typical walking levels. Although the exact strain gauge location in their study was not specified, the reported compressive strain values suggest they were placed on the dorsal‐lateral cortex, which corresponds to our *ex vivo* gauges on deer calcanei. The similarity in peak compressive strains between their startled sheep and our high‐load deer specimens suggests that both sets of data reflect high‐impact activities, such as kicking or bounding. In mule deer, such strains likely occur during their characteristic ‘pronk’ or ‘stott’ gaits, which do not occur in domesticated sheep (Lingle, 1992; Hildebrand & Goslow, 2001).

**Table 6:** Comparison of Lanyon's (1973, 1974) *in vivo* principal strains with *ex vivo* strains at comparable locations. The *in vivo* data were measured in walking sheep. *Ex vivo* data were measured under a 50 kg load applied to the calcaneus (with a fixed limb position) which corresponds to 0.3 times body weight, which is similar to *in vivo* load in walking sheep. * data for mule deer from Su *et al.* (1999) and Skedros *et al*. (2019); ** animal one (sheep) of Lanyon (1974); ++ animal S53 (sheep) of Lanyon (1973); + animal eight (sheep) of Lanyon (1974); # principal strain ratio = absolute value of: (minimum principal strain/maximum principal strain).

Gauge site	Loading procedure	Principal tensile strain (με)	Principal compressive strain (με)	Principal angle (°)	Principal strain ratio^#^
Dorsal‐ lateral	ex vivo*	171	−348	−85	2.0
	in vivo**	69	−200	−88	2.9
	*in vivo* ^++^	70	−200	–	2.9
Lateral	ex vivo*	348	−136	−28	0.4
	*in vivo* ^+^	300	−204	−30	0.7

In summary, the data from simulated loading experiments, and *in vivo* data from Lanyon (1974) and Skerry & Lanyon (1995), all support the conclusion that the plantar/dorsal tension/compression environment is a valid generalization of the habitual loading of the calcaneal shafts of these species.

### (2) Limitations of small sample sizes and desiccation effects on CFO

Despite the errors in Table 4 of McMahon *et al*. (1995), their text accurately summarizes some of the strain magnitude and mode differences described by Lanyon (1973). However, it is important to emphasize that they not only contested the simple ‘tension/compression bone’ concept with arguments based on their interpretation of Lanyon's (1973) single‐element strain gauge data but also based this refutation on their qualitative observations showing a lack of expected (strain‐mode‐specific) regional patterns of predominant CFO in a very small sample of sheep calcanei. Specifically, they did not find the expected plantar *versus* dorsal and regional CFO differences when a bone region (e.g. the artiodactyl calcaneus mid‐shaft) is relatively simply loaded (Fig. 4). A compelling argument can be made that their study lacked sufficient statistical power to test their null hypothesis. They only used five bones: two ‘sub‐adults’ and three ‘adults’, and specific ages were not stated. To investigate this, I performed retrospective sample size and power analyses (powerandsamplesize.com) using the CFO data from our study of CPL images from thin micro‐milled sections of plantar and dorsal cortices of domesticated sheep calcanei (Skedros *et al*., 2007a). That study included eight sub‐adult bones (8–10 months old) and five adult bones (2 years old) (Skedros *et al*., 2007a), with regional CFO differences summarized in Table 2. In the sub‐adult bones, the dorsal cortex CFO/WMGL was 110.7 ± 16.0 (mean ± SD) and plantar CFO/WMGL was 86.7 ± 14.6 (*P* < 0.05); where larger numerical values equate to more oblique‐to‐transverse CFO (‘compression‐adapted’ bone) and lower values to ‘tension adapted’ bone (Bromage *et al*., 2003; Skedros *et al*., 2009). Using the data from the eight sub‐adult bones, nine bones would be needed to detect this dorsal *versus* plantar difference with 95% statistical power (*α* < 0.05). The statistical power achieved with our original sample of eight sub‐adult bones was 93% (*α* < 0.05). For the five adult calcanei, we found dorsal CFO to be 94.8 ± 13.6 and plantar CFO to be 63.0 ± 12.2 (*P* < 0.01). Using these data from adults, four bones would be needed to detect this dorsal *versus* plantar difference with 95% statistical power (*α* < 0.05). The statistical power achieved with our original sample of five adult bones was 99% (*α* < 0.05). Applying the same approach to the sample sizes used by McMahon *et al*. (1995) for adult bones (two fresh and one dried) and *α* < 0.05, we can calculate a statistical power of only 79% for *N* = 2, representing only their fresh adult bones; 92% for *N* = 3, representing all of their adult bones; 97% for *N* = 4; and 99% for *N* = 5. If using a cutoff for adequate statistical power of ≥95% (which was achieved for *N* = 4 for our adult bone data), then a sample size of three bones will be insufficient for their hypothesis testing.

Of the five bones used by McMahon *et al*. (1995), only three (two adult, one sub‐adult) were fresh. The other two had been dried for many years before histological analysis, and all specimens were sourced from a New York City butcher (Timothy Bromage, personal communication). While Bromage reported that the thin sections ‘looked the same’ under CPL (implying that drying did not cause significant artifacts), there are no quantitative studies confirming an absence of effects of prolonged drying on CFO assessments. My observations indicate that a ‘smudged’ appearance can result when thinly sectioned bone specimens are dried and subsequently embedded (in polymethyl‐methacrylate) and viewed under polarized light. In addition, data from a microindentation study of mineralized turkey leg tendon (MTLT) show that the elastic modulus is on average 56% lower in the wet state than in a dried state (Spiesz, Roschger & Zysset, 2012). This is an important finding because the MTLT has been suggested as a possible calibration standard for quantitative CFO analyses in bone (Bromage *et al*., 2003; Spiesz, Kaminsky & Zysset, 2011; Warshaw *et al*., 2017). It is possible that this degradation is, in part, a consequence of desiccation‐related changes in CFO or other aspects of collagen organization (Bailey, Paul & Knott, 1998). If future studies confirm that prolonged drying impairs accurate CFO analysis, then the sample of McMahon *et al*. (1995) would be reduced to only three bones.

### (3) Limitations of the lifestyle of the animals sampled

McMahon *et al*. (1995) used domesticated sheep from the eastern USA; their animals likely had relatively sedentary lifestyles with limited long‐distance travel and no exposure to natural predation. Docility is known to be more pronounced in females than males (Hafez *et al*., 1962; Banks, 1964; Hulet, 1966; Grubb, 1974); three of their animals were female and the sex of the others was not reported. The lack of plantar/dorsal CFO patterns in their adult sheep calcanei could likely be attributed to the animals' docile lifestyles. Even within a single flock, individual variation of activity levels due to factors like age or sex may influence mechanical loading patterns. Data from Skedros *et al*. (2007a) on 13 sub‐adult and adult female sheep showed that one bone lacked the expected CFO differences, which we attributed to an age‐related reduction in activity. In addition to reduced activity levels, quantitative data show that limb joint excursions decrease with age in domesticated sheep (Faria *et al*., 2014).

The sheep used by Skedros *et al*. (2007a) were likely more physically active than those studied by McMahon *et al*. (1995), as our animals were sourced from a large open‐range sheep‐herding ranch (Julian Ranch, Kemmerer, Wyoming). Our sheep regularly travelled long distances (160–482 km) during seasonal grazing with the assistance of dogs – a practice resembling Spanish transhumance (Anonymous, 2009; de los Ángeles Ramo *et al*., 2018; Shaban & Benja, 2023) – and predator encounters (e.g. coyotes and cougars) likely increased their physical activity (Truman Julian, personal communication). Additionally, these sheep were raised primarily for wool production. These factors (Hargreaves & Hutson, 1997) suggest that the calcanei of the sheep in our study experienced more intense, variable, and frequent loading compared to those examined by McMahon *et al*. (1995). Natural variability in ambulatory activity/intensity, which can diminish the predictive value of predominant CFO for detecting habitual bending, may explain why some studies fail to find histomorphological evidence of bending adaptations in some bones (Goldman *et al*., 2003; Skedros, Demes & Judex, 2003b; Matsuo *et al*., 2019; Nguyen & Barak, 2020; Manandhar *et al*., 2023). Further research using animal models with better‐controlled activity levels will be needed to clarify how ambulatory activity influences bone organization (Rubin *et al*., 1996b; Thomas *et al*., 1996; Adams *et al*., 1997; Carlson & Judex, 2007; Meakin, Price & Lanyon, 2014; Vitienes *et al*., 2023).

Mule deer are highly migratory, with approximately 90% leaving their home ranges during a typical year (van de Kerk *et al*., 2021). The average migration for mule deer is reported to be 66 km (Berger, 2004), but extreme cases have been reported (up to 772 km) in Wyoming, USA (Joly *et al*., 2019; van de Kerk *et al*., 2021). In our mule deer studies, carcasses were obtained from northern Utah, a region with geographic similarities to many Wyoming deer habitats. Skedros *et al*. (2011d) compared *in vivo* microdamage in the calcanei of adult wild deer (*N* = 11) and domesticated sheep (*N* = 11) and found ample evidence of natural microdamage in deer but none in sheep. We speculated that longer migration distances might explain this difference. However, we later learned that the open‐range sheep we studied likely travelled at least three times farther annually than the deer, and hence that the higher prevalence of microdamage in deer calcanei compared to sheep calcanei (see Section V) may instead reflect their more dynamic locomotory behaviours, including running, jumping, darting, and pronking.

**Fig. 9:**
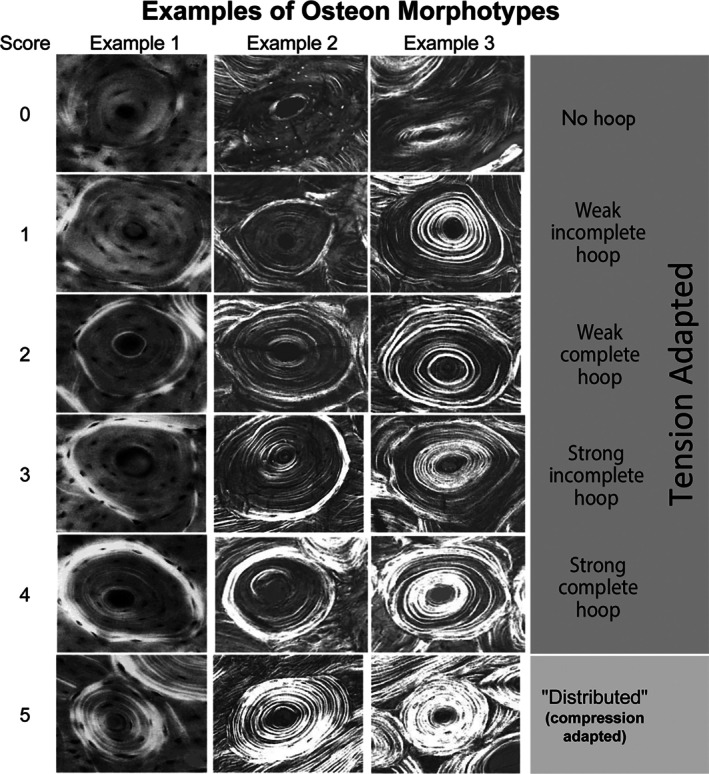
Examples of secondary osteon collagen/lamellar morphotypes. Example 1 images are from adult equine third metacarpals; examples 2 and 3 are from adult human and chimpanzee femora. Example 3 includes osteons with relatively greater brightness within the osteonal wall compared to those in examples 1 and 2. The ‘score’ at the left represents a numerical value given for each written description for each image in the far‐right column using methods described in Skedros *et al*. (2009). All circularly polarized light (CPL) images were obtained in the same imaging session, and specimens were undecalcified, unstained, and embedded in polymethyl‐methacrylate. Additional details regarding the presence and variations of brighter peripheral ‘hoops’ can be found in (Skedros *et al*., 2013a). Images reproduced with permission from John G. Skedros and Richard B. Martin.

**Fig. 10:**
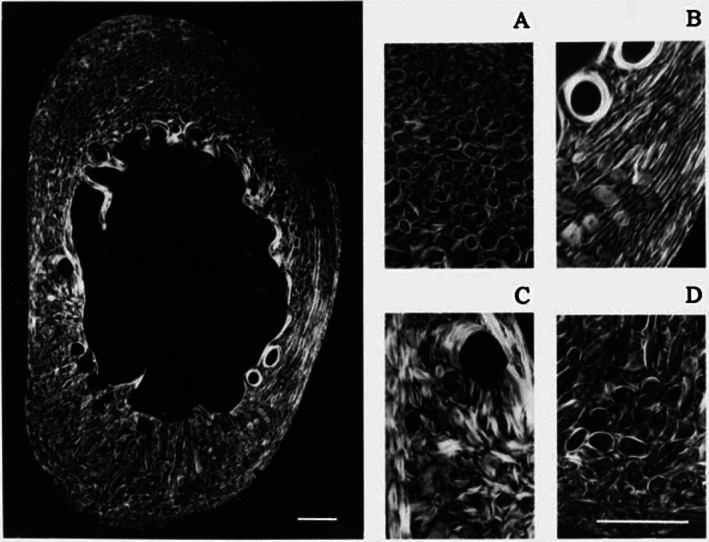
Circularly polarized light (CPL) images reproduced from McMahon *et al*. (1995). The image on the left shows a transverse thin section of a sub‐adult sheep calcaneus shaft (exact shaft location not specified). A–D are magnified regions from this section: (A) dorsal cortex, (B) plantar‐lateral cortex, (C) medial cortex, and (D) plantar cortex. The scale bars shown on the large image and image D represent 1 mm (A–C are at the same scale as D). Reproduced with permission of John Wiley and Sons, Inc.

In contrast to McMahon *et al*. (1995) we have reported quantitative data from CPL images showing regional differences in sheep and deer calcanei consistent with the plantar/dorsal tension/compression loading interpretation (Figs 4 and 10, Table 2) (Skedros *et al*., 2004, 2007a, 2013a). It is noteworthy that the CPL images illustrated by McMahon *et al*. (1995) for sub‐adult OVA6 show primarily ‘hoop’ or parallel‐fibered osteons (i.e. predominantly longitudinal, darker ‘tension‐adapted osteons’) in the dorsal and plantar cortices (compare Figs 9 and 10). Notably, in Fig. 10, the dorsal‐lateral cortex (i.e. upper right) of the larger image appears very dark. This could reflect non‐uniform grinding/polishing of the specimen, which artifactually darkens thinner regions of the specimen because the darker background predominates rather than the actual bone tissue. If correct, this might help to explain their general observation of darker grey levels in the ‘compression cortex’. We took care to avoid this artifact by using an ultramiller in our studies.

### (4) Effects of animal age

In addition to the small sample of fresh bones (*N* = 3) and the presumed docility of the animals used by McMahon *et al*. (1995), the disparity between their CPL observations and our CFO data may stem from the ages of their ‘sub‐adults’ and ‘adults’ compared to those in Skedros *et al*. (2007a). McMahon *et al*. (1995) neither specified animal age nor commented on the degree of growth plate closure. It is possible that their adults had recently matured, and their ‘sub‐adults’ were developmentally closer to younger sub‐adults, an age group in which regional strain‐mode‐related CFO variations in the dorsal *versus* plantar calcaneal cortices are often not statistically significant (Skedros *et al*., 2007a). This possibility that McMahon *et al*. (1995) used relatively young sub‐adults is supported by their own suggestion that the transverse (‘compression adapted’) CFO was paradoxically found in a ‘tension region’, citing the concentration of compact coarse cancellous bone in the region as a possible explanation.

Their reference to ‘compact coarse cancellous’ bone is important because this histology reflects recent incorporation of trabecular bone into the cortex during normal growth. This histological organization is generally absent in adult bones, especially in species that exhibit bone remodelling (i.e. secondary osteon formation) (Enlow, 1963; Currey, 1984; Cadet *et al*., 2003; Legendre & Botha‐Brink, 2018). We did not observe compact coarse cancellous bone in any of our older sub‐adult bones (Skedros *et al*., 2007a). However, if the histomorphology is developmentally constrained (i.e. not strongly dependent upon strain stimuli) as suggested by McMahon *et al*. (1995), then their observation might suggest that tissue‐level constraints during growth, especially rapid bone formation, could produce CFO patterns that appear puzzling or regionally generic, especially in the earlier stages of bone growth.

In bone, modelling drift describes the continuous process where bone is deposited on one surface while simultaneously resorbed from an opposing surface, leading to changes in bone shape, size, curvature, and position over time. This asymmetric process is distinct from bone remodelling, which replaces old bone with new bone on the same surface (and typically *via* osteon formation) (Skedros *et al*., 2011a). It is known that rapid bone formation and/or modelling drifts during growth can confound interpretations of functional significance of regional CFO variations in the cortices of sub‐adult limb bones (Skedros & Kuo, 1999; Lee, 2004; Skedros *et al*., 2004, 2007a). Increasing evidence suggests that, in some cases, correspondence of regional CFO variations with load history might be absent or difficult to detect until the later stages of growth, when osteonal remodelling is more prevalent (Skedros & Kuo, 1999; Warshaw *et al*., 2017). Warshaw *et al*. (2017) also argued that consideration must be given to the type of histology present (e.g. fibrolamellar, parallel‐fibered, ‘hybrid’, lamellar, compacted coarse cancellous, Sharpey fibre, and secondary osteonal) (see also Prondvai, Stein & Ricqlès, 2014). Section V discusses the mechanical implications of differences in the amount of regional circumferential lamellar bone in sheep *versus* deer calcanei. The potential roles played by different collagen/matrix ‘motifs’ (e.g. twisted plywood *versus* orthogonal plywood designs) should also be explored, as these may be the underlying physical bases of variations in collagen organization in different primary and non‐primary histological types (Giraud‐Guille, 1988; Yamamoto *et al*., 2012; Skedros *et al*., 2016). Further explorations are important because most studies that evaluate patterns of predominant CFO in bone cortices in the context of load history focus on regionally ‘averaged’ birefringence (i.e. image grey level) differences in polarized light.

### (5) The ‘tension‐resistance priority hypothesis’ and the ‘shear‐resistance priority hypothesis’

To align their CFO and histomorphological observations better with Lanyon's (1973) *in vivo* data from single‐element gauges, McMahon *et al*. (1995) speculated that some of Lanyon's gauge placements were imprecisely reported. For example, in the two animals (S34 and S35) with gauges on the dorsal‐lateral (‘compression’) and plantar‐lateral (‘tension’) cortices, they proposed that the plantar‐lateral gauge might have actually been placed more dorsally, closer to the ‘compression region’. This would help reconcile their CPL findings of both bright and dark CFO in this area, as more oscillatory strain patterns (near the neutral axis) could explain such mixed collagen orientations. However, Lanyon's illustrations show that none of these gauges were placed on the mid‐lateral cortex. To explain their paradoxical finding of predominantly longitudinal (‘tension adapted’) CFO in the dorsal‐lateral (compression) cortex, McMahon *et al*. (1995) presented the tension‐resistance priority hypothesis (TRPH): ‘the occurrence of even a small magnitude of tensile strain acting on a localized bone volume may be sufficient to stimulate the formation of longitudinal collagen fibres, even when the same bone region is undergoing greater compressive strain during another phase of the locomotor cycle’ (McMahon *et al*., 1995, p. 157). Their argument was supported by mechanical testing data showing that bone tissue is generally less resistant to tension than compression, an idea that is strongly supported by recent literature.

The TRPH has received some attention. For example, Raguin & Drapeau (2020) hypothesized that because double zonal osteons are also defined by a change in collagen fibre orientation, their formation could be regulated by differences in mechanical loads. In support of this idea, they noted that McMahon *et al*. (1995) ‘proposed that the magnitude [and mode] of strain may also play an important role on preferred collagen orientation. From the sheep calcaneus, the authors showed that low levels of localized tensile strain are sufficient to change the collagen orientation, even if the bone is predominantly subjected to compressive load.’ (Raguin & Drapeau, 2020, p. 600). Although this idea might seem plausible, it is based on a flawed interpretation of ‘strain reversals’ in the sheep calcaneus model (Tables 3 and 4; Fig. 8), as argued above. McMahon *et al*. (1995) also proposed the ‘holistic response’ hypothesis to explain unexpected CFO patterns in their sheep calcanei and other bones, including the macaque (*Macaca mulatta*) mandible and human tibia. The holistic response hypothesis posits that CFO, in combination with other microstructural and macrostructural features, functions to contribute to the overall maintenance of Young's modulus (tissue stiffness). The holistic response seems to be embodied in a more comprehensive, integrated hypothesis proposed by Schlecht & Jepsen (2013). Their study examined functional relationships between bone robustness [(total cross‐sectional area)/(bone length)], cortical tissue mineral density, and cortical area in various limb bones from young adult African Americans. They found strong support for the idea that variation in these integrated traits allows bone to maximize tissue stiffness and minimize mass, ensuring sufficient strength and stiffness for routine loading, regardless of phenotype. If this integrative model, or a similar one that also considers strength and energy absorption (Ritchie *et al*., 2008; Burr, 2011; Zimmermann, Busse & Ritchie, 2015; Skedros *et al*., 2024b), is validated by controlled biomechanical experiments, it may help explain paradoxical regional CFO patterns. For example, it could clarify why darker osteons can appear unexpectedly in the CPL images from the dorsal and plantar cortices of a sub‐adult calcaneus (Fig. 10, top *versus* bottom cortex).

**Fig. 11:**
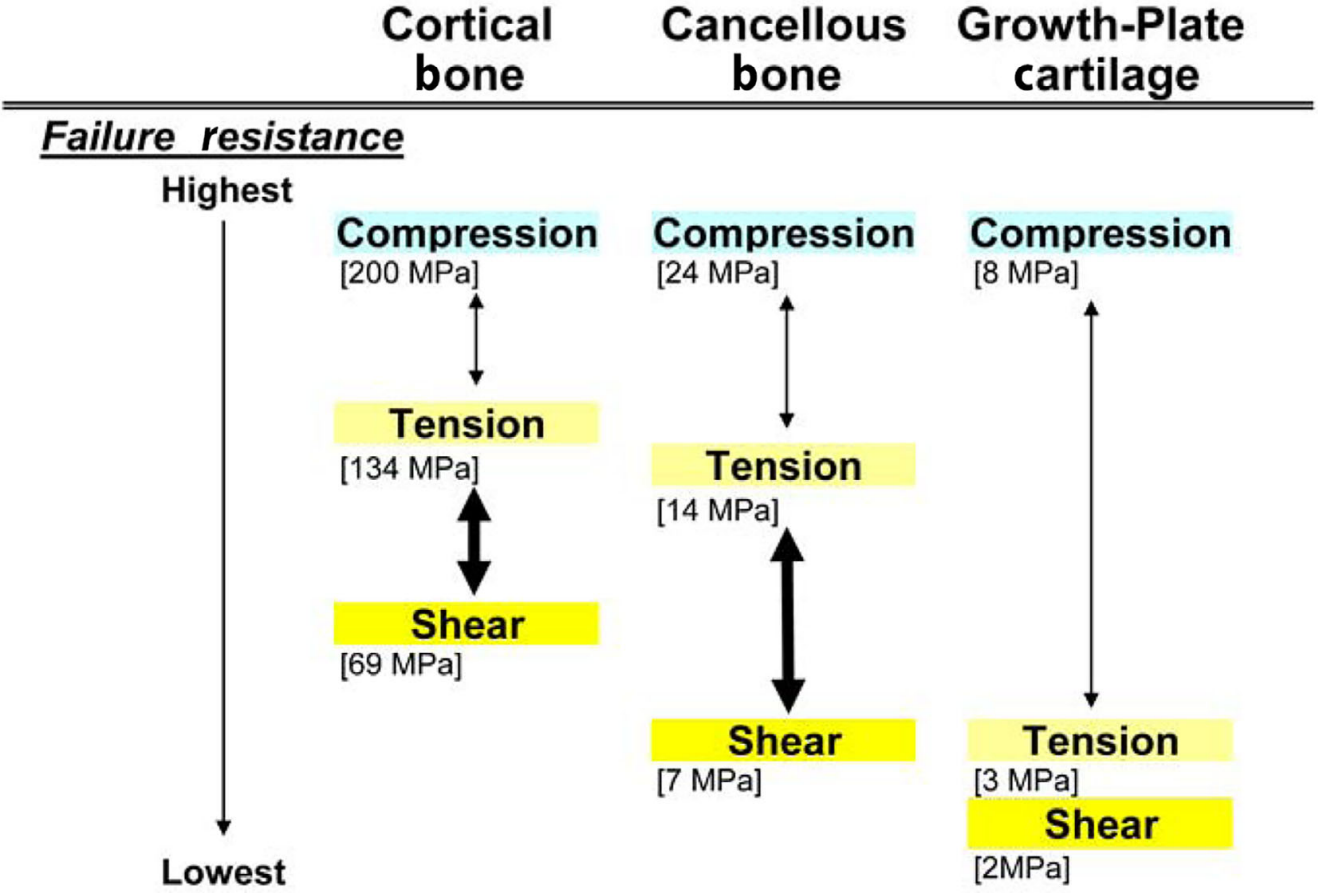
Diagrammatic representation of the ‘shear‐resistance priority hypothesis’. For cortical bone, the values are from bovine bone (Cowin, 1989). Cancellous bone values are estimated from Keaveny *et al*. (2001) for bovine bone using strength anisotropy ratios (longitudinal ÷ transverse strength) and bone volume fractions between 0.3 and 0.5. For cartilage, the compression value is estimated from human articular cartilage (Yamada, 1970). Values for tension and shear are from bovine tibia growth plates (Williams *et al*., 1999). Reproduced from Skedros & Baucom (2007) with permission of John G. Skedros.

While the TRPH may apply in some cases (particularly when shear strains are minimal), it falls short for most bone adaptation studies because it neglects shear, the ‘third’ and often most damaging strain mode. In many studies of load‐history‐related bone material adaptation, shear strains are not adequately considered when compared to the other two strain modes. In an *ex vivo* study examining strains during typical functional loading of adult mule deer calcanei, Su *et al*. (1999) specifically countered this bias by focusing their analysis on how shear strains vary with respect to changing loads and with respect to tension and compression in the main cortical regions (dorsal, plantar, and neutral axis). They reported that maximum shear strains typically exceeded the principal compression and tension strains in the dorsal and plantar cortices. In a subsequent study using the same bones, this interpretation was revised by using a more rigorous ‘expression’ of shear values (Skedros *et al*., 2019). When considering material‐axis shear strains, which are more biologically relevant than maximum shear strains (Carter, 1978), Skedros *et al*. (2019) showed that the deer calcaneus is primarily loaded in this more accurate expression of shear only in the medial and lateral cortices. This is expected in the neutral axis location of a unidirectional end‐loaded short‐cantilevered beam (Young, 1989, p. 97). Additionally, Skedros *et al*. (2019) confirmed that compression is the dominant strain mode in the dorsal cortex and tension is the dominant strain mode in the plantar cortex at peak stance of walking and running, and also with off‐axis loads that simulated less‐typical locomotor activities. However, because material‐axis shear strains were shown to have relatively greater prevalence in the plantar ‘tension region’ cortex (when compared to the dorsal and medial/lateral cortices), it was suggested that the artiodactyl calcaneus might be best viewed as a ‘tension‐shear/compression bone’ with respect to the plantar/dorsal strain distribution. Because shear is the most deleterious among the three strain modes (Burstein *et al*., 1972; Reilly & Currey, 2000; Turner, Wang & Burr, 2001; Hiller *et al*., 2003; Taylor *et al*., 2003; Skedros & Baucom, 2007), the ‘shear‐resistance priority hypothesis’ was introduced, supplanting the TRPH (Warshaw *et al*., 2017; Daegling, 2022) (Fig. 11).

**Fig. 12:**
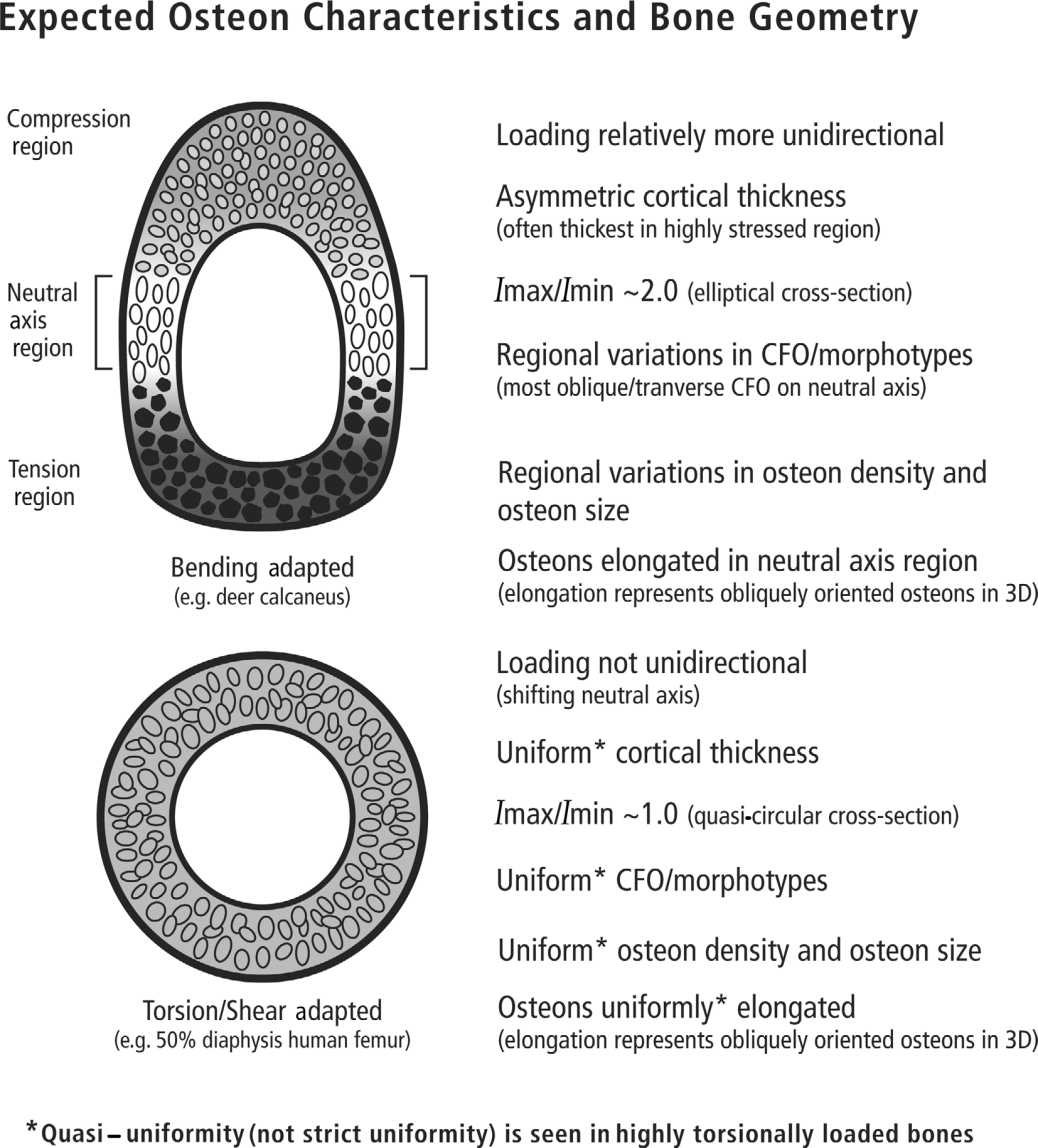
Expected osteon characteristics and bone geometry in response to bending and torsion/shear. Top: strain‐related regional material variations in a bone undergoing habitual unidirectional bending (low‐complexity loading) showing regionally prevalent/predominant strain modes (compression (top), shear (middle), and tension (bottom) as observed in artiodactyl (sheep and deer) calcaneus models. Bottom: lack of regional material variations in a bone subject to habitual torsion/multi‐directional loading, such as the mid‐diaphyseal region of human or chimpanzee femora (Goldman *et al*., 2003; Beckstrom *et al*., 2010). The regional strain‐related microstructural variations for the non‐uniform strain distribution in the top drawing are not necessary due to the more uniform strain distribution experienced by the bone section in the bottom drawing. *I*max/*I*min represents the distribution of mass in accordance with the second moment of area (*I*) of the tubular cross‐section of the bone. *I*max and *I*min are, respectively, the greatest and least bending resistance of the cross section. Therefore, in the top drawing *I*max is 2, and this is the direction of the long axis of the elliptical cross‐section. CFO, collagen fibre orientation. Reproduced from Keenan *et al*. (2017) with permission of John G. Skedros.

It is important to emphasize that in bones subjected to significant shear strains from prevalent torsion (i.e. high‐complexity loading), regional histomorphological adaptations to localized prevalent/predominant strain modes, including shear, do not occur. This is because torsional/multi‐directional loading produces diffusely distributed shear strains, minimizing biologically meaningful regional differences in strain prevalence (Skedros *et al*., 2009). In such cases, shear is likely the most biomechanically relevant strain mode, and adaptation manifests as uniform matrix organization; for example, relatively consistent oblique‐to‐transverse CFO patterns across the entire cross section, as seen in torsionally loaded sheep tibiae and turkey ulnae (Skedros & Hunt, 2004; Skedros *et al*., 2009). This concept is illustrated in Fig. 12. In contrast to the sheep tibia (high‐complexity loading), artiodactyl calcanei are relatively simply loaded, where shear strains are comparatively more localized along the medial‐lateral (neutral axis) region (Skedros *et al*., 2019). The higher prevalence/predominance of shear strains in this region might explain the distinct CFO patterns and osteon morphotypes compared to adjacent ‘compression’ and ‘tension’ cortices (Fig. 4), a pattern also seen in other limb bones habitually loaded with relatively unidirectional bending (Skedros *et al*., 2009, 2011b, 2013a). Additional discussion on the role of shear in mediating cortical and trabecular adaptations in the artiodactyl calcaneus and human femoral neck can be found in Skedros & Baucom (2007), Skedros *et al*. (2019, 2023) and von Kroge *et al*. (2022).

## III. USE OF CFO AND OSTEON COLLAGEN/LAMELLAR MORPHOTYPES TO INTERPRET LOAD HISTORY

In order to evaluate critically the strengths and weaknesses of using CFO patterns to infer habitual ‘tension/compression loading’ in any bone region (i.e. net tension on one cortex and net compression on the opposing cortex), it is important to discuss the strengths and weaknesses of this type of retrospective analysis (Skedros *et al*., 2013a).

### (1) Strain modes, predominant CFO patterns and related bone matrix characteristics

**Fig. 13:**
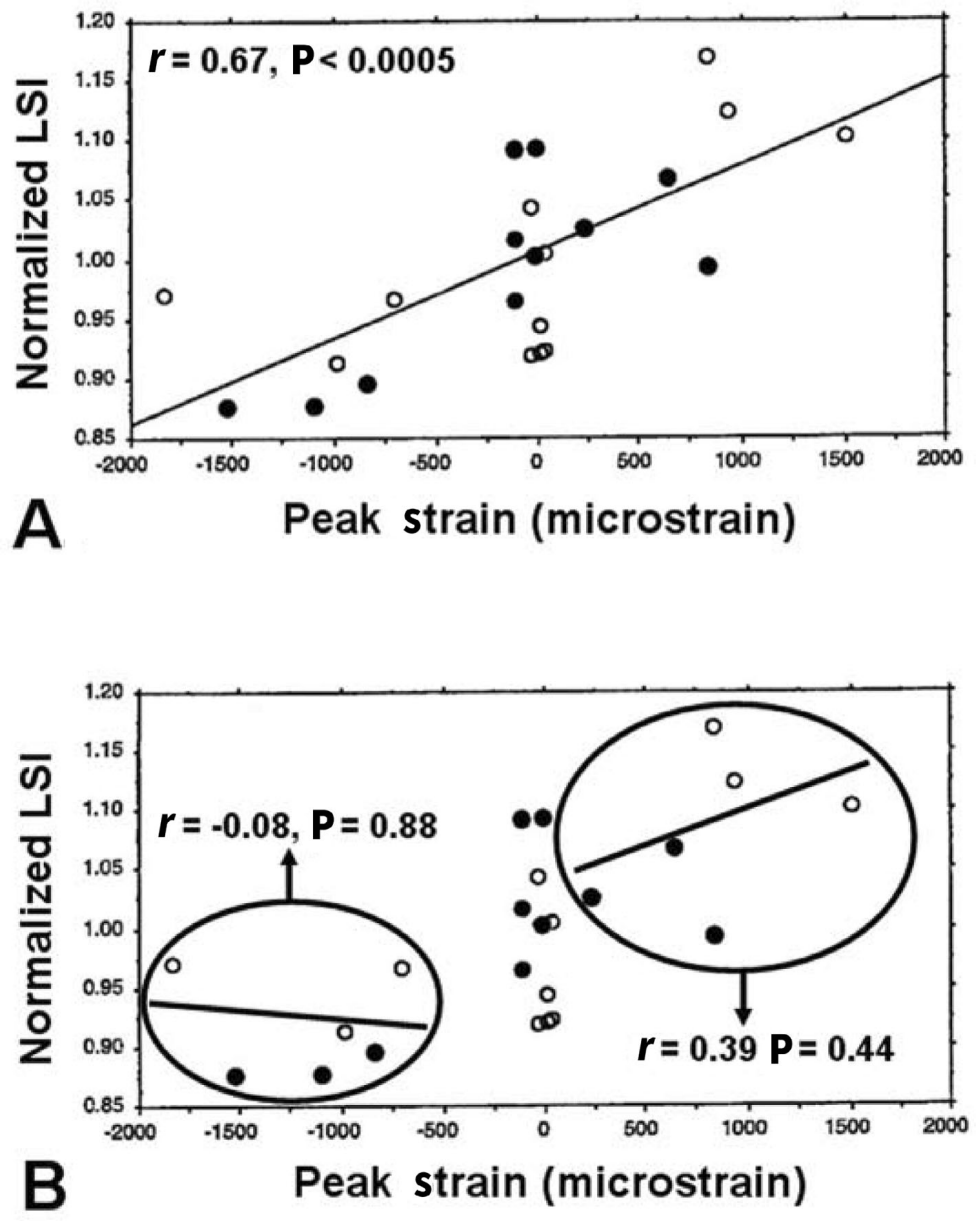
Modified version of fig. 8 from Takano *et al*. (1999). (A) Original data showing a significant correlation between longitudinal structure index (LSI) and peak strain measurements in radii from dogs in sham‐operated (black circles) and baseline (white circles) groups. LSI represents the predominant collagen fibre orientation (CFO) of the bone tissue with respect to the long axis of the bone (Martin *et al*., 1996; Bromage *et al*., 2003). (B) Ellipses and Pearson least‐squares correlation coefficients added to the original graphic, showing no significant correlations between CFO [obtained from circularly polarized light (CPL) images] and strain magnitude when analysed separately for regions experiencing compression (left with negative microstrain values) or tension (right with positive microstrain values). Modified from Takano *et al*. (1999) and reproduced with permission from John Wiley and Sons, Inc.

In cortical regions of many limb bone diaphyses, regional variations in predominant CFO and associated secondary osteon collagen/lamellar ‘morphotypes’ (Fig. 9) are strongly correlated with the strain‐mode distribution of habitual bending, i.e. net tension on one cortex and net compression on the other (Boyde & Riggs, 1990; Riggs *et al*., 1993; Skedros & Kuo, 1999; Skedros *et al*., 2009, 2011b, 2013a; Keenan *et al*., 2017). Similar strain‐mode‐specific patterns are evident in the dorsal and plantar trabecular tracts of adult mule deer calcanei (J. G. Skedros, J. T. Cronin, C. S. Mears & B. W. Richards, in preparation). Data from both cortical and trabecular bone strongly suggest that there is a causal relationship between predominant strain mode and CFO, at least in the context of simple unidirectional bending. However, this causal relationship has been clearly shown in only one prospective controlled study that examined only cortical bone (Takano *et al*., 1999). They showed that an experimentally induced change in the strain‐mode distribution (i.e. a ‘strain‐mode reversal’) caused strain‐mode‐specific matrix formation; namely, more oblique‐to‐transverse CFO in the new ‘compression region’ and more longitudinal CFO in the new ‘tension region’. Additional evaluation of their data shows that the experimentally induced strain‐mode‐specific matrix changes do not correspond to the trans‐cortical variations in strain magnitude but appear to be responses to the strain mode that prevails across the entire cortex (Fig. 13). It is important to note that the differences between the CPL/CFO data in the two ellipses in Fig. 13B trend toward being significantly different (*P* = 0.07) (i.e. this suggests a causal strain‐mode effect) but did not reach statistical significance likely due to insufficient sample size (Charles H. Turner, personal communication). By contrast, the acoustic data reported by Takano *et al*. (1999) are statistically significant (*P* < 0.05), showing a strong relationship between predominant CFO and strain mode. Note that LSI and predominant CFO are inversely related (i.e. lower LSI values correspond to more oblique‐to‐transverse CFO) (Fig. 13). This contrasts with our approach and that of other investigators (Bromage *et al*., 2003; Goldman *et al*., 2003; Warshaw *et al*., 2017) where CFO is based on WMGLs with, for example, higher grey levels corresponding to more oblique‐to‐transverse CFO.

A collaborative retrospective study also found strong correlations between strain‐mode‐specific CFO variations and both acoustic microscopy and CPL data in dorsal and plantar cortices of adult mule deer calcanei (Skedros *et al*., 2006b). They also reported an extraordinary finding: mineral crystallite orientation and predominant CFO appear to be dissociated. This suggests that extrafibrillar mineral can preferentially orient in a mechanically relevant direction (e.g. with respect to principal compression strains) that differs from the preferred CFO orientation optimized for principal tension strains. This implies that mineral crystallite orientation in the dorsal ‘compression cortex’ of the artiodactyl calcaneus plays a more significant role in accommodating compression, whereas in the plantar cortex, predominant CFO may have greater relevance in accommodating tension. The observation that mechanically relevant dissociation of mineral crystallites from preferred orientation of collagen fibrils can occur has been largely unrecognized in the current literature [see Stockausen *et al*. (2021) and references therein].

The artiodactyl calcaneus model seems well‐poised for the examination of the functional relevance of differences in distributions and preferred orientations of mineral crystallite aggregates known as ‘tesselles’ (McKee, Buss & Reznikov, 2022) and described as ‘mineral prolate ellipsoids’ (Micheletti *et al*., 2022). This would require imaging technologies that provide very high resolution, such as focused ion beam‐scanning electron microscopy (FIB‐SEM), submicron computed tomography (μCT), and electron energy loss spectroscopy (EELS) (Binkley *et al*., 2020; McKee *et al*., 2022; Micheletti *et al*., 2022; Rodriguez‐Palomo, Østergaard & Birkedal, 2024; Weiner & Shahar, 2024).

### (2) Regional variations in CFO

Although regional patterns of predominant CFO are strong predictors of habitual unidirectional bending, they are not always reliable indicators of load history (Kalmey & Lovejoy, 2002; Skedros *et al*., 2003b, 2008; Lee, 2004; de Margerie *et al*., 2005; Main, 2007; Skedros, 2011; Skedros & Doutré, 2019). There are exceptions that reduce both the sensitivity and specificity of CFO patterns. For example, in the third metacarpal of fruit bats (*Glossophaga soricina*), reversals in bending loads during flight produce nearly equal tension and compression strain magnitudes and durations on opposing cortices (Swartz & Middleton, 2008; Skedros & Doutré, 2019). Regional variations in predominant CFO might also be unreliable for interpreting load history where early bone growth is rapid, such as in radius bones of young sheep (Skedros & Kuo, 1999) and horses (Riggs *et al*., 1993), where expected regional CFO differences fail to develop in early ontogeny.

To evaluate better the reliability of regional variations in predominant CFO and related bone material characteristics (Table 2) as indicators of specific loading histories (e.g. simple bending *versus* torsion), it is important to establish their diagnostic accuracy more rigorously. This can be accomplished by amassing a large body of data from prospective experimental studies across various bones and species, allowing calculation of sensitivity and specificity values. These studies could examine bone loading in several contexts, including: (*i*) bones with natural distributions of specific strain modes in spatially segregated regions (e.g. canine and sheep radius, and sheep calcaneus); (*ii*) experimentally induced changes in the strain‐mode distribution of those same bones (e.g. as in Takano *et al*., 1999); and (*iii*) experimentally induced changes in the strain environment designed to obscure regional strain‐mode‐specific adaptations (e.g. inducing torsion/shear to an additional experimental group in Takano *et al*., 1999). Sensitivity would be calculated as the proportion of true positives correctly identified by predominant CFO; for example, correctly identifying a region experiencing net tension (*via in vivo* strain gauges) as having more longitudinal CFO (seen as darker WMGL). Specificity would be the proportion of true negatives that are correctly identified by predominant CFO; for example, regions not under net tension showing predominant CFO that is not relatively more longitudinal (i.e. not showing darker WMGL). Using data amassed from many species, these analyses would be conducted similarly to how sensitivity and specificity are calculated for clinical diagnostic testing (Altman & Bland, 1994a,b; Rosner, 2006). Initial analyses should be conducted in established models with naturally occurring osteonal remodelling and well‐documented changes in strain distributions, such as the canine and sheep radius, and turkey ulna (*Meleagris gallopavo domesticus*) (Lanyon *et al*., 1979, 1982; Rubin *et al*., 1996b; Srinivasan & Gross, 1999; Takano *et al*., 1999; Meakin *et al*., 2014).

### (3) Load‐complexity categories

There are cases where regional CFO patterns appear to have little to no correlation with a load history presumed to involve directionally consistent bending. We suggest that in many of these cases, the loading is more varied than commonly assumed, placing the bone region in a ‘higher complexity’ load category (Table 7). Examples include the mid‐shaft regions of adult human and chimpanzee (*Pan troglodytes*) femora, where ambient bending is thought to occur, but torsion is likely of greater biomechanical consequence; this is because torsion‐induced loading causes shear that obscures expected variations in regional CFO patterns (Goldman *et al*., 2003; Skedros & Baucom, 2004; Skedros *et al*., 2011b; Keenan *et al*., 2017; Manandhar *et al*., 2023) (Fig. 12). Another notable example has been described in the radius of adult goats (*Capra hircus*) (Main, 2007; see also Main *et al*., 2021) where there was a lack of the ‘expected’ regional tension‐ and compression‐adapted CFO patterns as seen in our studies of sheep radii (Skedros & Kuo, 1999; Skedros *et al*., 2009). These ‘unexpected’ results in goats likely reflect their more variable locomotory behaviours compared to adult sheep (Moreno *et al*., 2008; McGuigan *et al*., 2009; Leung *et al*., 2015; Lloveras *et al*., 2022), including climbing trees and other activities rarely exhibited by domesticated sheep (Delibes, Castañeda & Fedriani, 2017; Barr, 2020; Daegling, 2022). Accordingly, in Table 7, the goat radius is in a higher complexity load category (‘High’) than the sheep radius (‘Moderate’).

**Table 7:** Working hypothesis for the four load‐complexity categories based on neutral axis (N.A.) rotation or other criteria during the middle portion of typical load phase. **‡** These studies, despite not reporting neutral axis rotation, contain strain gauge or related data that allow reasonable inference of load‐complexity category. **§** (with colour highlight), these are bones or bone regions classified in more than one category due to factors such as changes in habitual activity during ontogeny (e.g. domesticated turkey ulna), habit (e.g. goat *versus* sheep radius, **), or volitional activity (e.g. humans or racing horses). * Generally high shear strain can confound these categorizations in some cases (e.g. chicken tarsometatarsus). This might help explain why the human femoral neck is in the high complexity category (Skedros *et al.*, 2023). Updated from versions in Keenan *et al.* (2017), Cronin & Skedros (2024).

Complexity category	Examples	
**Low** (N.A.: <10° rotation) (**Tension and compression minimally overlap; shear is localized near N.A**.)	**1. artiodactyl and perissodactyl calcanei**	
		(Orders Artiodactyla and Perissodactyla)
		(Lanyon, 1974‡; Su *et al*., 1999; Skedros *et al*., 2019)
	**2. potoroo calcaneus** (*Potorous tridactylus*)	
		(Biewener *et al*., 1996‡)
	**3. chicken tarsometatarsus** (**TMT**) (*Gallus gallus domesticus*)*	
		(Judex *et al*., 1997b; Skedros *et al*., 2003b‡)
	**4. iguana tibia** (*Iguana iguana*)	
		(Blob & Biewener, 2001)
	**5. alligator tibia** (*Alligator mississippiensis*)	
		(Blob & Biewener, 2001)
**Moderate A** (N.A.: 10° – 20° rotation)	6. dog, sheep and horse radii §, **	
		(*Canis lupus familiaris*); (*Ovis aries*); (*Equus caballus*)
		(Carter *et al*., 1980‡; Lanyon *et al*., 1982‡, Coleman *et al*., 2002; Takano *et al*., 1999‡)
	**7. dog ulna** (*Canis lupus familiaris*)	
		(Carter *et al*., 1980‡)
	**8. macaque ulna** (*Macaca mulatta*)	
		(Demes *et al*., 1998; Skedros *et al*., 2003b‡)
	**9. macaque tibia** (*Macaca mulatta*)	
		(Demes *et al*., 2001)
**Moderate B** (N.A.: 20° – 45° rotation)	**10. human fibula** (*Homo sapiens*)	
		(Lambert, 1971; Thomas *et al*., 1995; Weaver & Skedros, 2016)
	**11. human tibia** (*Homo sapiens*) **§**	
		(Lanyon *et al*., 1975‡; Burr *et al*., 1996‡; Milgrom *et al*., 2000‡; Peterman *et al*., 2001; Yang *et al*., 2014)
	**12. human femur proximal diaphysis** (*Homo sapiens*) **§**	
		(Cochran *et al*., 1980‡; Aamodt *et al*., 1997‡; Skedros *et al*., 1999‡; Skedros & Baucom, 2007‡; Skedros *et al*., 2012a‡)
	**13. chimpanzee femoral neck** (*Pan troglodytes*) **§**	
		(Kalmey & Lovejoy, 2002‡; Skedros *et al*., 2008‡)
	**14. dog tibia** (*Canis lupus familiaris*)	
		(Bouvier & Hylander., 1984‡; Yoshikawa *et al*., 1994)
	**15. dog proximal femur** (*Canis lupus familiaris*)	
		(Page *et al*., 1993‡; Shahar *et al*., 2003‡)
	**16. dog femur mid‐diaphysis** (*Canis lupus familiaris*)	
		(Schatzker *et al*., 1980‡; Szivek *et al*., 1992; Shahar *et al*., 2003‡)
	**17. horse third metacarpal** (*Equus caballus*) **§**	
		(Gross *et al*., 1992; Skedros *et al*., 1996)
	**18. sheep metatarsal** (*Ovis aries*)	
		(Lieberman *et al*., 2004)
	**19. armadillo femur** (*Dasypus novemcinctus*)	
		(Copploe *et al*., 2015)
	**20. bat metacarpal** (*Glossophaga soricina*)	
		(Swartz & Middleton, 2008‡; Skedros & Doutré, 2019)
	**21. immature turkey ulna** (*Meleagris gallopavo domesticus*) **§**	
		(Skedros & Hunt, 2004)
	**22. chicken tibiotarsus** (*Gallus gallus domesticus*)	
		(Biewener *et al*., 1986‡; Biewener & Bertram 1993b‡; Vitienes *et al*., 2023‡)
	**23. tegu lizard femur** (*Tupinambus merianae*)	
		(Sheffield *et al*., 2011)
**High** (N.A.: >45° rotation) (**Tension and compression overlap extensively; shear is relatively more diffusely distributed across the cortex when compared to other categories**.)	**24. human tibia** (**in some athletes**) (*Homo sapiens*) **§**	
		(Lanyon *et al*., 1975‡; Burr *et al*., 1996‡; Milgrom *et al*., 2000‡; Peterman *et al*., 2001; Yang *et al*., 2014)
	**25. human femur mid‐diaphysis** (*Homo sapiens*) **§**	
		(Duda *et al*., 1998‡; Goldman *et al*., 2003‡; Drapeau & Streeter, 2006‡; Edwards *et al*., 2008‡)
	**26. human femoral neck** (*Homo sapiens*)	
		(Pidaparti & Turner, 1997‡; Skedros *et al*., 1999‡; Skedros & Baucom, 2007‡; Edwards *et al*., 2008‡; Kersh *et al*., 2018; Deng *et al*., 2021)
	**27. chimpanzee femoral neck** (*Pan troglodytes*) **§**	
		(Kalmey & Lovejoy 2002‡; Skedros *et al*., 2008‡)
	**28. horse third metacarpal** (*Equus caballus*) **§**	
		(Skedros *et al*., 2006a; Rubin *et al*., 2013‡)
	**29. goat radius** (*Capra hircus*) §, **	
		(Main & Biewener, 2004; Main, 2007; Moreno *et al*., 2008‡)
	**30. sheep tibia** (*Ovis aries*)	
		(Lanyon & Bourn, 1979‡; Lieberman *et al*., 2004; Gautier *et al*., 2000‡)
	**31. free‐flying bat humerus** (*Glossophaga soricina*)	
		(Swartz *et al*., 1992‡; Swartz & Middleton, 2008‡; Skedros & Doutré, 2019)
	**32. mature turkey ulna** (*Meleagris gallopavo domesticus*) **§**	
		(Rubin & Lanyon, 1985; Skedros & Hunt, 2004)
	**33. emu femur and tibiotarsus** (*Dromaius novaehollandiae*)	
		(Main & Biewener, 2007‡)
	**34. pigeon humerus** (*Columba livia*)	
		(Biewener & Dial, 1995; Skedros & Doutré, 2019)
	**35. chicken femur** (*Gallus gallus*)	
		(Carrano & Biewener, 1999‡; Skedros, 2002‡)
	**36. alligator femur** (*Alligator mississippiensis*)	
		(Blob & Biewener, 1999; Blob & Biewener, 2001; Lee, 2004‡)
	**37. iguana femur** (*Iguana iguana*)	
		(Blob & Biewener, 2001)
	**38. opossum femur** (*Didelphis virginiana*)	
		(Butcher *et al*., 2011; Gosnell *et al*., 2011)
	**39. river cooter turtle femur** (*Pseudemys concinna*)	
		(Butcher & Blob, 2008; Aiello *et al*., 2013)

In the ‘High’ complexity category, loading causes the greatest variability in strain distributions, which usually result in prevalent torsion‐induced strains. Torsional loading produces shear, tension, and compression strains that are spatially and temporally diffusely/uniformly distributed. This contrasts with the strain distributions in lower‐complexity load categories where bending loads are comparatively unidirectional, causing dominant tension, compression, and shear in generally mutually exclusive regions (e.g. as seen in sheep calcanei and radii) (Keenan *et al*., 2017; Skedros *et al*., 2019) (Fig. 12). Deer and sheep calcanei are good examples of bones in the ‘Low’ complexity category (Table 7), making them potential control bones for comparative studies (Sinclair *et al*., 2013; Skedros *et al*., 2013a, 2024a) as discussed in Section VII.1. For example, in their study of the microstructural differences between the cranial and caudal cortices of the upper humerus of white‐tailed deer (*Odocoileus virginianus*) calcanei, Nguyen & Barak (2020) used the artiodactyl calcaneus essentially as a control bone. They referred to our prior studies reporting clear patterns of strain‐mode/magnitude‐related microstructural adaptations between the tension‐ and compression‐loaded cortices of artiodactyl calcanei (Table 2). This allowed them to infer the presence of microstructural adaptations for habitual net tension in the cranial cortex *versus* net compression in the caudal cortex, resulting from the presumed habitual bending environment of the deer proximal humerus; as also suggested for humeri of 13–33‐month old Soay sheep at mid shaft (Becker *et al*., 2020). Unfortunately, this causal relationship remains uncertain due to lack of *in vivo* or *ex vivo* strain data from the humeri of any sheep or deer species.

In bones that experience relatively consistent bending patterns, biomechanically significant but infrequent events – known as high‐complexity loading – can occur naturally (e.g. darting, climbing, and jumping) (Skedros & Hunt, 2004; Skedros *et al*., 2006a, 2019; Moreno *et al*., 2008). These perturbations are often quasi‐idiosyncratic, occurring more frequently in some individuals than others within the same species. The confounding effects of such variability on interpretations of limb bone adaptability have been considered in wild primates (Carlson *et al*., 2008), humans, and domesticated turkeys (Adams *et al*., 1997). This issue is particularly relevant for humans; for instance, relatively sedentary healthy adults who regularly participate in impact sports like basketball, soccer, tennis, or running (Burr *et al*., 1996; Haapasalo *et al*., 2000; Milgrom *et al*., 2003; Agostinete *et al*., 2016; Maillane‐Vanegas *et al*., 2020; Saers *et al*., 2021). Although it is unclear at what threshold loads become sufficiently varied to obscure regional bending‐related CFO patterns *via* modelling/remodelling, brief episodes of otherwise infrequent loads can be highly potent stimuli for bone adaptation (Rubin *et al*., 1995, 1996a; Skerry & Lanyon, 1995).

### (4) Ontogenetic emergence of strain‐mode‐related osteon collagen/lamellar ‘morphotypes’

The available data suggest that dorsal *versus* plantar differences in osteon morphotypes do not emerge in deer and sheep calcanei until the animals approach skeletal maturity, when bone growth slows and regional osteon variations are produced as a consequence of sufficiently prevalent or intense strain‐related stimuli (Reilly & Currey, 1999; Lieberman *et al*., 2003; Skedros *et al*., 2011d). In this context, it can be hypothesized that when the strains are not sufficient for evoking ‘compression‐adapted osteons’, relatively darker osteons form due to a ‘default’ collagen orientation that is less oblique‐to‐transverse (Main *et al*., 2021). This idea is consistent with a CPL image in Riggs *et al*. (1993) of a thin mid‐shaft transverse section of a young horse (foal) radius, which showed relatively uniform dark grey levels across the entire section, including the cranial ‘tension region’ and caudal ‘compression region’ cortices. As also shown in their CPL images of equine radii, distinct cranial *versus* caudal strain‐mode‐specific CFO differences emerge at later stages of development when secondary osteons began to appear. Similar findings of more longitudinal CFO in younger sub‐adults *versus* more oblique‐to‐transverse CFO in adult bones were observed in an ontogenetic series of sheep radii (Skedros & Kuo, 1999). In both species, darker, more uniform CPL grey levels in the younger bones might represent a generic or default CFO in the absence of ‘sufficient’ region‐specific strain‐mode‐related stimuli (Skedros *et al*., 2016).

In contrast to CFO data from young horses, we noted significantly brighter grey levels in CPL (hence more oblique‐to‐transverse CFO) in fetal and newborn deer and sheep calcanei (note that this is woven bone) (Fig. 4) when compared to those from older sub‐adults and adults (Skedros *et al*., 2004, 2007a). Relatively more oblique‐to‐transverse CFO has also been suggested to reflect the default matrix design in an experimental study conducted in cortices of canine limb bones (Mehraban Alvandi *et al*., 2013), which examined polarized images of transversely sectioned fourth metacarpals of seven adult dogs. Osteons that formed during the immobilization period showed overall brighter grey levels (oblique‐to‐transverse CFO) compared to control animals (normal loading) (*P* < 0.02). The authors concluded that, in the absence of mechanical loading, oblique‐to‐transverse CFO represents the default pattern for bone collagen orientation (the statistical power of their analysis was >99%). Additionally, in a study comparing human femoral cortical bone from one fetus, one newborn, and four 2–14 year olds, Zimmerman *et al*. (2019) found that predominant CFO became more longitudinal (i.e. darker in CPL) with increasing age.

In view of these conflicting observations, investigators should be cautious when considering what might constitute a ‘default’ CFO, as multiple possibilities likely exist. These may depend on aspects of programmed development, poorly understood loading conditions, or other unrecognized factors. For example, differences in a ‘default’ predominant CFO may reflect differences in the rate of osteogenesis, which could result in the formation of different histological ‘types’, spanning from woven bone to various types of lamellar and fibrolamellar bone (de Margerie *et al*., 2004; Lee, 2004; Skedros & Hunt, 2004; Reisinger, Pahr & Zysset, 2011; Almany Magal *et al*., 2014). A more comprehensive understanding of what constitutes a ‘default’ predominant CFO, and the reasons for its variability, is clearly needed.

## IV. USE OF THE ARTIODACTYL CALCANEUS TO STUDY TRABECULAR ARCHITECTURE AND MICRODAMAGE

### (1) The trajectorial hypothesis

**Fig. 14:**
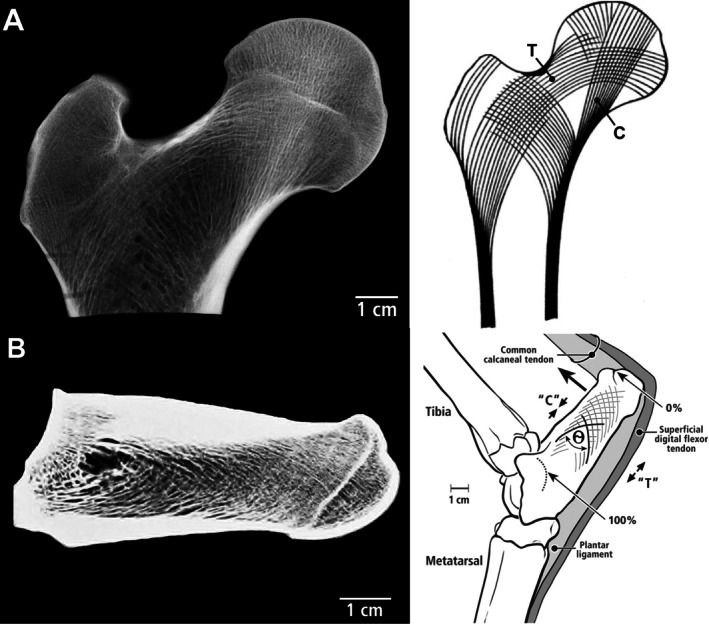
(A) Radiograph of a thin frontal section the femur of a 42‐year‐old human male (left) and von Meyer's (1867) drawing showing accurate two‐dimensional depictions of arched trabecular tracts (right). T = tension tract; C = compression tract. (Skedros & Brand, 2011; von Meyer, 2011). (B) Radiograph of a thin sagittal section of an adult mule deer calcaneus in lateral‐to‐medial view (left) and drawing of an adult mule deer calcaneus showing stylized arched trabecular tracts (right). The trabecular arches intersect in the middle portion of the shaft at approximately orthogonal angles, in accordance with simple unidirectional loading and the trajectorial theory. T = tension, C = compression. Reproduced from Skedros *et al*. (2023) with permission of John G. Skedros.

Skedros & Baucom (2007) studied trabecular bone morphology of sheep and deer calcanei in the context of Wolff's trajectorial hypothesis of trabecular architecture, which predicts orthogonal trabecular intersection angles between tension and compression stress trajectories. This arrangement is reflected in the arched trabecular tracts shown in Figs 1 and 14. They used a two‐dimensional analysis of intersection angles between the compression and tension trabecular tracts in lateral radiographs of these bones to test a prediction based on a basic tenet of Wolff's law: that there is an exact mathematical relationship of trabecular architecture to the ratio of maximal performance to minimal weight (Cowin, 2001; Skedros & Brand, 2011; Wood *et al*., 2019). Notably, the main trabecular intersections seen in lateral radiographs of deer and sheep calcanei intersect at ~90° angles, consistent with a trajectorial arrangement seen in simple unidirectional bending.

In a computed tomography (CT) study of trabecular architectural characteristics of sheep and deer calcanei, Sinclair *et al*. (2013) did not quantify trabecular intersection angles, although the authors recognized relatively simple *versus* more complex loading conditions for the proximal *versus* distal calcaneal shaft. In a study of mineral content of the dorsal and plantar cortices, and the relative amount of trabecular bone along the calcaneal shaft, Skedros *et al*. (1994a) described the proximal half of the calcaneal shaft as the ‘pure beam’ portion and the distal half as the ‘mixed beam’ portion (i.e. where loading is more complex). It remains unclear whether trabeculae are generally aligned with principal loading directions, supporting the trajectorial hypothesis, or off‐axis, which would be inconsistent with that hypothesis (Pidaparti & Turner, 1997; Skedros & Baucom, 2007; Wood *et al*., 2019). Robust finite element models will be needed to determine principal loading directions more precisely. Three‐dimensional imaging studies are also needed to compare trabecular intersection angles between the more beam‐like proximal portion of the calcaneal shaft where orthogonal intersection angles are expected *versus* the less beam‐like distal (apophyseal) end, where non‐orthogonal intersection angles are likely prevalent.

In a microcomputed tomography (micro‐CT) study of trabecular architectural motifs in the head, neck, and proximal metaphyseal region of adult human femora, Reznikov *et al*. (2016, p. 65) suggest that the trabecular bone resembles ‘…tensegrity structures—where lightweight, resilient and stable tetrahedron‐based shapes contribute to strain redistribution amongst all the elements and to collective impact dampening.’ This intriguing concept deserves additional study using the artiodactyl calcaneus model and in various regions of bones subjected to differing load histories. They reported prevalent non‐orthogonal angles, including angles of ~70°, similar to those we identified in our two‐dimensional analysis of intersecting trabecular tracts in the human femoral neck (Skedros & Baucom, 2007) (Fig. 14A). Reznikov *et al*. (2015) also noted a similar non‐orthogonal relationship between trabeculae alignment and predominant CFO within the trabeculae. Although their two studies (Reznikov *et al*., 2015, 2016) did not cite Pidaparti & Turner's (1997) two‐dimensional finite element analysis, which concluded that non‐orthogonal inter‐trabecular angles reduce deleterious shear stresses during multidirectional loading, their findings do support this important biomechanical implication. These and other studies of trabecular bone architecture, especially those employing analytical models to explore Wolff's trajectorial hypothesis and the law derived from it (e.g. Boyle & Kim, 2011; Giorgio *et al*., 2021, 2023) would benefit by using a simply loaded bone verified by *in vivo* strain analysis, like the artiodactyl calcaneus, as a comparative control.

### (2) Microdamage and mechanisms of 3D osteon formation

In bone, naturally forming microdamage can be strain‐mode specific, meaning that the size, shape, prevalence, and three‐dimensional (3D) alignment of microdamage entities (e.g. microcracks) can differ in regions dominated by tension, compression, or shear (Boyce *et al*., 1998; Reilly & Currey, 1999; George & Vashishth, 2005; Skedros *et al*., 2011d; Wolfram *et al*., 2016). Because osteons repair microdamage, it is reasonable to assume that the 3D morphology of microdamage could influence the size, shape, and 3D orientation of the osteons formed in response (Martin & Sobelman, 2004; Martin, 2007; Martinéz‐Reina *et al*., 2014). In a study of microdamage in adult artiodactyl calcanei, we hypothesized that strain‐mode‐specific microdamage would result in strain‐mode‐specific differences in the osteons that repair them (Skedros *et al*., 2011d). More specifically, this could account for regional variations in osteon cross‐sectional size, shape (e.g. more elliptical shape in obliquely oriented osteons), length, and 3D orientation (Fig. 5). Testing this hypothesis will require controlled experiments and high‐resolution 3D analyses of calcaneal cortices from experimental *versus* control groups. It is also possible that regional variations in CFO are influenced by the 3D orientation of osteons. However, available indirect data suggest that the organization of the tissue within an osteon is more strongly influenced by the strain environment in which it forms rather than by the osteon's 3D migration path (Ascenzi & Bonucci, 1972; Ascenzi, 1988; Mehraban Alvandi *et al*., 2013; Hennig *et al*., 2015; Skedros *et al*., 2016).

Based on my unpublished observations of epifluorescent images of India‐ink‐stained mid‐sagittal sections of adult deer calcanei, there is evidence that osteons in the dorsal and plantar cortices follow somewhat similar arching patterns, resembling theoretical stress trajectories. The idea that a secondary osteon's 3D orientation is significantly influenced by the dominant load direction is supported by modern analytical studies that consider cellular activities and the physiological milieu of the nascent resorption spaces that ultimately lead to osteon formation (Smit, Burger & Huyghe, 2002; Burger, Klein‐Nulend & Smit, 2003; Klein‐Nulend, Bacabac & Mullender, 2005). The trajectorial hypothesis has been considered by earlier investigators in the context of osteon alignment; namely, that osteons course obliquely in a way that seems to approximate stress trajectories in accordance with the ‘tension/compression environment’ of bones under habitual bending. For example, Murray (1936) describes Benninghoff's observations of longitudinal sections of the dolphin radius. This bone, presumed to experience habitual bending, exhibits arched trabecular patterns that seem to approximate stress trajectories produced by this bending (as described previously by Wilhelm Roux in the late 1890s). Murray (1936) concluded that the preferential curvilinear orientations of slit marks made with an awl in the cortical bone are aligned in continuation with trabeculae of the cancellous bone. This led Benninghoff to propose that osteons align in accordance with Wolff's trajectorial theory of trabecular bone architecture, where the dominant trabecular patterns are regarded as ‘accurate materialisations of the lines of principal force in a homogeneous body of the same form as the bone and stressed in the same way’ (Murray, 1936, p. 135). Thus, it is speculated that cortical bone forms, at least in part, by the ‘condensation of the materialized trajectories where … the osteones [sic] must, according to Benninghoff, be trajectorial materializations in the same sense as the trabeculae of the spongiosa [cancellous bone]’ (Murray, 1936, p. 135). This idea echoes the origin of Wolff's law of the functional adaptation of bone, which can be traced to the seminal observation that arched trabecular patterns in a sagittally sectioned human metatarsal resemble the theoretical stress trajectories in a similarly shaped and similarly loaded cantilevered beam (von Meyer, 1867, 2011; Skedros & Brand, 2011) (Fig. 1B). Among all vertebrate bones, the artiodactyl calcaneus is perhaps the best natural example of a cantilevered beam‐like trajectorial structure (Skedros & Baucom, 2007), as recognized by Lanyon (1974) for sheep calcanei.

Heřt, Fiala & Petrtýl (1994) and Petrtýl, Heřt & Fiala (1996) considered the idea that osteons align with principal strains, concluding that osteons approximate directions of the principal stresses, which were estimated using an analytical model. They found that the osteons at the femoral mid‐diaphysis deviate by ~6–10° with respect to the bone's long axis; this off‐axis alignment closely corresponds to the orientation of their calculated principal stresses. In other human bones, the osteons were seen also to align obliquely with respect to their longitudinal axes: tibiae 6–8°, humeri 12–15°, radii ~8°, and ulnae ~8°. However, these results are inconsistent with *in vivo* strain data and two‐dimensional microscopic observations obtained from adult sheep tibiae reported by Lanyon & Bourn (1979). They found that principal strains deviated with respect to the tibia's long axis by ~28.8° in the cranial cortex and ~ 22.4° in the caudal cortex. In contrast to the relatively close correspondence of osteon orientation and principal strain direction shown by Heřt *et al*. (1994) and Petrtýl *et al*. (1996), the osteons observed by Lanyon & Bourn (1979) in their two‐dimensional images appeared to deviate by ~11.5° in the cranial cortex and ~ 9.5° in the caudal cortex with respect to the long axis of the bone. Notably, these studies are severely limited by a lack of 3D analyses and did not use bones with simple and well‐documented load histories.

An analytical study by Martinéz‐Reina *et al*. (2014) provided observations that raised further questions regarding the relationship between osteon alignment and predominant strain directions. They noted that the images published by Heřt *et al*. (1994) and Petrtýl *et al*. (1996) showed wide variability in the length of the vascular canals that was not mentioned in those publications. Based on their analysis, Martinéz‐Reina *et al*. (2014) concluded that the prior observations of these investigators from the 1990s are likely underestimates and that osteons actually deviate from the long axis of the diaphysis by values 2–3.5 times greater than previously reported. These large differences suggest that conventional expectations of a ‘trajectorial relationship’ in osteon orientations may be flawed and that further work is needed to advance our current understanding of the mechanisms that govern the formation and preferential migration of the basic multicellular units (BMUs) that form secondary osteons, and detailed 3D studies of osteon networks in limb bones from various species that represent a spectrum of the complexity of loading (see Table 7). These issues would be best examined in models that have relatively simple load histories and are amenable to *in vivo* strain measurement and experimental perturbation. A suitable well‐described experimental model could be the sheep ankle (hock) external‐fixator model (Skerry & Lanyon, 1995) in which the ankle region can be immobilized, thus effectively removing natural strain from the calcaneus. Loss of ‘strain tropism’ in this model means that the osteons would not be expected to migrate in the hypothesized directions of stress trajectories. *In vivo* and *ex vivo* studies of this model thus have potential for advancing understanding of the mechanisms that mediate bone microstructural adaptation beyond what is possible in other, more extensively studied animal models (Lanyon, 1980; Lanyon *et al*., 1982; O'Connor *et al*., 1982; Skerry *et al*., 1990; Rubin *et al*., 1996b; Srinivasan & Gross, 1999; Hsieh & Turner, 2001; Robling *et al*., 2001; Herman *et al*., 2010; Meakin *et al*., 2014; Baumann *et al*., 2015). These other models lack the myriad regional variations in material organization found in artiodactyl calcanei (Table 2), reducing their translational research value for understanding the mechanobiology of appendicular bones of humans and other primates. Samples obtained from cortical regions of experimentally manipulated sheep calcanei (e.g. unloaded) could be evaluated using synchrotron radiation‐based micro‐CT scanning for the 3D visualization of osteon structure in both control and treated specimens (Varga *et al*., 2013; Maggiano *et al*., 2016; Pratt *et al*., 2018; Maggiano, Maggiano & Cooper, 2021; Loundagin *et al*., 2024). Subsequent thin sectioning of the specimens could be used to analyse regional patterns of predominant CFO and secondary osteon collagen/lamellar morphotypes (Skedros *et al*., 2009). Notably, previous studies exploring these issues were not designed to discern causal relationships (Cohen & Harris, 1958; Tappen, 1977; Lanyon & Bourn, 1979; Heřt *et al*., 1994; Petrtýl *et al*., 1996; Robling & Stout, 1999; Stout *et al*., 1999; Martinéz‐Reina *et al*., 2014; Hennig *et al*., 2015).

Micro‐CT images obtained by D.M. Cooper from one skeletally mature mule deer calcaneus allow viewing the 3D alignment and interconnections of secondary osteon canals (Haversian canals) with respect to the longitudinal direction of the shaft. Transverse section images were used to create Z‐stack ‘videos’ from a cylindrical volume of interest from the mid‐cortical region of the dorsal and plantar cortex at mid shaft. The Z‐stack video reconstructions of the osteonal canals in these two cortical locations are provided as online Supporting Information in Videos S1 and S2. The general methods for obtaining and reconstructing these serial transverse micro‐CT images have been described previously (Cooper *et al*., 2003; Skedros *et al*., 2014). These videos show that osteon canals typically course obliquely in the sagittal plane in the dorsal and plantar cortices, but it is currently unclear if they approximate the course of the trabecular arches (hence approximating theoretical stress trajectories). This uncertainty reflects the fact that the orientations of these videos with respect to the orientation of the trabecular tracts of the single sampled deer calcaneus were not recorded with certainty. While these preliminary observations of osteon obliquity are compelling, as they may resemble curvilinear stress trajectories, this interpretation remains speculative pending further investigation.

## V. FROST'S MECHANOSTAT HYPOTHESIS AND LAMELLAR BONE FORMATION

**Fig. 15:**
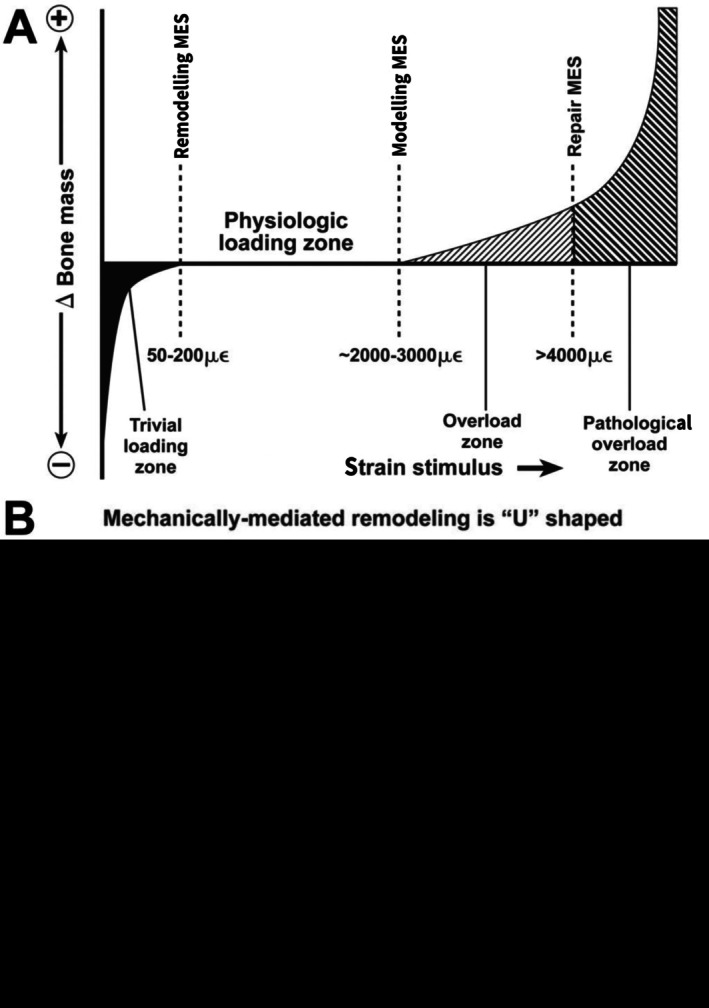
Strain magnitude thresholds of Frost's mechanostat hypothesis for bone modelling and remodelling. The figure illustrates the four mechanical usage zones defined by the mechanostat hypothesis, highlighting the differing thresholds for bone modelling and remodelling responses. Below the minimum effective strain (MES) of remodelling (low or trivial loading zone), strains are low, and bone remodelling is activated. Above the remodelling MES but below the modelling MES (the physiological loading zone), remodelling activity is relatively repressed and is also under the influence of hormonal and metabolic influences. Above the modelling MES, lamellar bone is gained through increased modelling. Above the repair MES (pathological overload zone), new woven bone is added rapidly to bone surfaces; this is neither modelling nor remodelling but probably represents a repair reaction. Reproduced from Skedros *et al*. (2001) with permission of John G. Skedros.

Skedros *et al*. (2001) argue that structural and material differences between the dorsal and plantar cortices of sheep and deer artiodactyl calcanei (Table 2) closely align with predictions of Frost's mechanostat hypothesis (Frost, 1987) for the relationship between modelling/remodelling activities during the growth and maintenance of a limb bone under habitual bending. In that study, we emphasized the apparent relationship between these activities and presumed strain‐magnitude‐based thresholds (as depicted in Fig. 15). The mechanostat hypothesis assumes that strain thresholds control the activation of modelling and remodelling, and that these thresholds likely differ between the dorsal and plantar cortices. Limited experimental data support this hypothesis (Hsieh *et al*., 2001; Skerry, 2006; Miller *et al*., 2021, 2024). The artiodactyl calcaneus model holds promise for evaluation of specific predictions stemming from the mechanostat hypothesis, given that there are well‐described experimental methods that allow unloading to excessive loading of this bone (Skerry & Lanyon, 1993, 1995; Thomas *et al*., 1996). Indeed, sheep are generally considered appropriate models for translational studies in bone health and disease in humans (e.g. osteoporosis and glucocorticoid abnormalities) (Kennedy *et al*., 2009; Healy *et al*., 2010; Cabrera *et al*., 2018; Banstola & Reynolds, 2022). A major shortcoming of the mechanostat hypothesis is that it does not account sufficiently for the roles of systemic factors, such as hormones, on bone remodelling (Lee *et al*., 2003; Oheim *et al*., 2012). Studies of bone remodelling have been conducted using limb bones of female sheep, both with and without perturbation of normal oestrogen levels, typically achieved *via* ovariectomy (Kennedy *et al*., 2009; Healy *et al*., 2010). Altered hormone levels can enable evaluation of modelling and remodelling dynamics in calcanei subjected to different load conditions (Newman, Turner & Wark, 1995; Skerry & Lanyon, 1995; Thomas *et al*., 1996).

The artiodactyl calcaneus model is also well suited for investigating the potential mechanical relevance of regional variations in the amount of circumferential lamellar bone in wild animals, where strains are likely higher compared to domesticated animals. Mechanical testing of bovine bone has shown that secondary osteonal bone is weaker than primary bone (Currey, 1975, 1984; Carter, Hayes & Schurman, 1976). However, in these studies, the primary bone referenced is not primary circumferential lamellar bone but is primary fibrolamellar (also known as plexiform) bone, which is a combination of parallel‐fibred bone and lamellar bone (de Ricqlès *et al*., 1991; Liu, Wagner & Weiner, 2000). In contrast to fibrolamellar bone, circumferential lamellar bone that forms the outer layers of the cortices of the limb bones of many mammals functions primarily to provide resistance to bending and torsional forces. These forces are most deleterious at the peripheral regions of the bone, where strains are highest. The mechanical resilience of circumferential lamellar bone is due to its layered structure and optimized interlamellar patterns of collagen fibre orientation (Currey, 1984). During fracture healing and normal growth, lamellar bone replaces the initially fast‐forming and rather weak ‘woven bone’, which is prevalent during early ontogeny of sheep and deer calcanei (Skedros *et al*., 2004, 2007a). Controlled experiments using the rat tibia four‐point loading model (Turner *et al*., 1994) show that lamellar bone formation is greatest in the regions of bone exposed to the highest strains. By contrast, woven bone formation occurs independent of the magnitude of bone strain and was stimulated as much by pressure applied to the periosteum (sham loading) as by applied bending strains. Therefore, Turner *et al*. (1994, p. 94) concluded that ‘… lamellar bone formation was driven either directly or indirectly by strains in the bone tissue.’ Considering that wild deer likely rely on frequent rapid acceleration to escape predators, there could be periods of very high strain that cause damage requiring repair. This idea was indirectly supported by an analysis of *in vivo* microdamage in the calcanei of adult wild deer (*N* = 11) and domesticated sheep (*N* = 11), which found relatively extensive natural microdamage in the deer but none in the sheep (Skedros *et al*., 2011d).

These observations prompted me to examine thin sections from my archive of adult sheep and deer calcanei using CPL microscopy, specifically in the mid‐to‐proximal shaft region where load sharing from the trabecular bone is minimal. For 15 specimens from each species, I found that compared to sheep calcanei, deer calcanei exhibited: (*i*) a clear predominance of regional lamellar bone in the peripheral cortex, particularly along the high‐strain regions of the outer dorsal and dorsal‐lateral cortices; and (*ii*) a lamellar pattern was observed in nearly 80% of the deer specimens *versus* 20% of the sheep specimens. This discrepancy may reflect exceptionally high strains in certain deer; this intriguing observation requires controlled experiments to account for possible confounding influences of growth modelling, animal age, and physical activity levels. Nevertheless, published quantitative data in sheep and deer calcanei indicate that, from the sub‐adult to adult stages, modelling drift mainly occurs on the dorsal cortex in the dorsal direction (Skedros *et al*., 2001, 2007a). It is not surprising that classical circumferential lamellae are absent along the outer cortical margin of the plantar cortex (Skedros *et al*., 2001) due to the attachment of the plantar ligament along that region. Notably, the difference in the average thickness of the circumferential lamellar bone between the outer dorsal/compression cortex of immature (*N* = 19) and mature (*N* = 15) deer calcanei is not significantly different (Skedros *et al*., 2001). These measurements have not yet been made in sheep calcanei.

## VI. REGIONAL MECHANICAL PROPERTIES

### (1) Strain‐mode‐specific and non‐strain‐mode‐specific mechanical testing

Differences in mineral content and material organization between the dorsal and plantar cortices of deer calcanei significantly influence mechanical properties in their predominant/habitual loading mode (compression *versus* tension) (Skedros *et al*., 2024a). One mechanical testing approach to discern these differences is called ‘strain‐mode‐specific (S‐M‐S) testing’ (Skedros *et al*., 2006a, 2024a,b; Manandhar *et al*., 2023). It is applicable to many mammalian limb bones because some degree of unidirectional bending is common (Rubin & Lanyon, 1984a; Biewener *et al*., 1986; Biewener & Bertram, 1993a,b; Demes *et al*., 2001; Lovejoy *et al*., 2002; Lieberman *et al*., 2003; Moreno *et al*., 2008; Milne, 2016). Table 8 summarizes the mechanical properties of bone specimens obtained from the dorsal (compression) cortex and plantar (tension) cortex of adult mule deer calcanei and subjected to independent S‐M‐S testing (highlighted in Table 8) and non‐strain‐mode‐specific (non‐S‐M‐S) testing (non‐highlighted in Table 8) (Skedros *et al*., 2024a). Notably, as revealed by the more physiologically relevant S‐M‐S tests, all three energy absorption parameters [i.e. elastic (pre‐yield), plastic (post‐yield), and total] were approximately three times greater in compression tests of the dorsal cortex compared to its tension tests. Unexpectedly, tension testing of the plantar/tension cortex did not show greater energy absorption (pre‐, post‐, and total) compared to compression testing of that same cortex. In fact, compression testing of the dorsal cortex outperformed tension testing of the plantar cortex in four of the six mechanical properties. The only metric where the plantar cortex was superior was elastic modulus, indicating that its tissue is stiffer in tension than the dorsal cortex is in compression. At first glance, these results suggest that during functional loading, the dorsal cortex protects the plantar cortex, as it is stronger and can absorb more energy prior to failure. The (putative) protection function of the dorsal cortex thereby helps to ensure an adequate safety factor for the whole bone. However, when considering the two load‐sharing tension members (ligament and tendon) that course along the plantar cortex, surprisingly there is equivalent load sharing between the dorsal cortex and the plantar fibro‐osseous cortex (Skedros *et al*., 2023, 2024a). This indicates that the plantar cortex is not generally deficient (as seems to be suggested by most of the S‐M‐S data in Table 8) when considered in this load‐sharing context. These considerations are likely applicable to other similarly loaded bone regions in other species and possibly to bones with moderate‐complexity loading (Table 7).

**Table 8:** Mechanical properties of the dorsal and plantar cortices of adult deer calcanei. Values are means and values in parentheses are standard deviations. Data are from Skedros *et al.* (2023, 2024a). Grey highlight indicates strain‐mode‐specific (S‐M‐S) data; non‐highlighted values indicate non‐S‐M‐S data. S‐M‐S D:P ratios appear in the far‐right column. C = compression; T = tension; D = dorsal; P = plantar. ^‡^ *P* ≤ 0.05 and ^Tr^ 0.05 < *P* ≤ 0.09 for the paired comparison represented by the ratio.

Mechanical property	Load mode	Dorsal calcaneus	C:T ratio	Plantar calcaneus	C:T ratio	D:P ratio	S‐M‐S D:P ratio
**Elastic modulus** [GPa]	**C**	**15.0**		**7.7**		**1.9** ^**‡**^	
		(2.3)		(2.3)		[15.0/7.7]	**0.7** ^**‡**^
	**T**	**20.7**	**0.7** ^**‡**^	**20.4**	**0.4** ^**‡**^	**1.0**	[15.0/20.4]
		(1.7)	[15.0/20.7]	1.9	[7.7/20.4]	[20.7/20.4]	
**Yield stress** [MPa]	**C**	**187.1**		**130.3**		**1.4** ^**‡**^	
		(12.2)		(22.1)		[187.1/130.3]	**1.5** ^**‡**^
	**T**	**122.4**	**1.5** ^**‡**^	**121.4**	**1.1** ^**‡**^	**1.0**	[187.1/121.4]
		(10.7)	[187.1/122.4]	(9.2)	[130.3/121.4]	[122.4/121.4]	
**Ultimate stress** [MPa]	**C**	**198.8**		**137.1**		**1.5** ^**‡**^	
		(13.2)		(20.1)		[198.8/137.1]	**1.6** ^**‡**^
	**T**	**126.6**	**1.6** ^**‡**^	**128.2**	**1.1** ^**‡**^	**1.0**	[198.8/128.2]
		(13.0)	[198.8/126.6]	(11.1)	[137.1/128.2]	[126.6/128.2]	
**Pre‐yield energy** [J/m^3^]	**C**	**1817.5**		**1536.9**		**1.2** ^**‡**^	
		(150.3)		(332.4)		[1817.5/1536.9]	**3.1** ^**‡**^
	**T**	**591.5**	**3.1** ^**‡**^	**586.4**	**2.6** ^**‡**^	**1.0**	[1817.5/586.4]
		(78.2)	[1817.5/591.5]	(55.7)	[1536.9/586.4]	[591.5/586.4]	
**Post‐yield energy** [J/m^3^]	**C**	**1409.7**		**760.6**		**1.9**	
		(527.4)		(422.6)		[1409.7/760.6]	**1.3**
	**T**	**504.6**	**2.8** ^**Tr**^	**1062.2**	**0.7**	**0.5**	[1409.7/1062.2]
		(367.4)	[1409.7/504.6]	(1049.1)	[760.6/1062.2]	[504.6/1062.2]	
**Total energy** [J/m^3^]	**C**	**3227.2**		**2297.5**		**1.4** ^**Tr**^	
		(433.1)		(456.7)		[3227.2/2297.5]	**2.0** ^**‡**^
	**T**	**1096.1**	**2.9** ^**‡**^	**1648.7**	**1.4** ^**‡**^	**0.7**	[3227.2/1648.7]
		(424.7)	[3227.2/1096.1]	(1086.3)	[2297.5/1648.7]	[1096.1/1648.7]	

### (2) Asymmetric distribution of cortical thickness and trabecular bone to reduce fracture risk

**Fig. 16:**
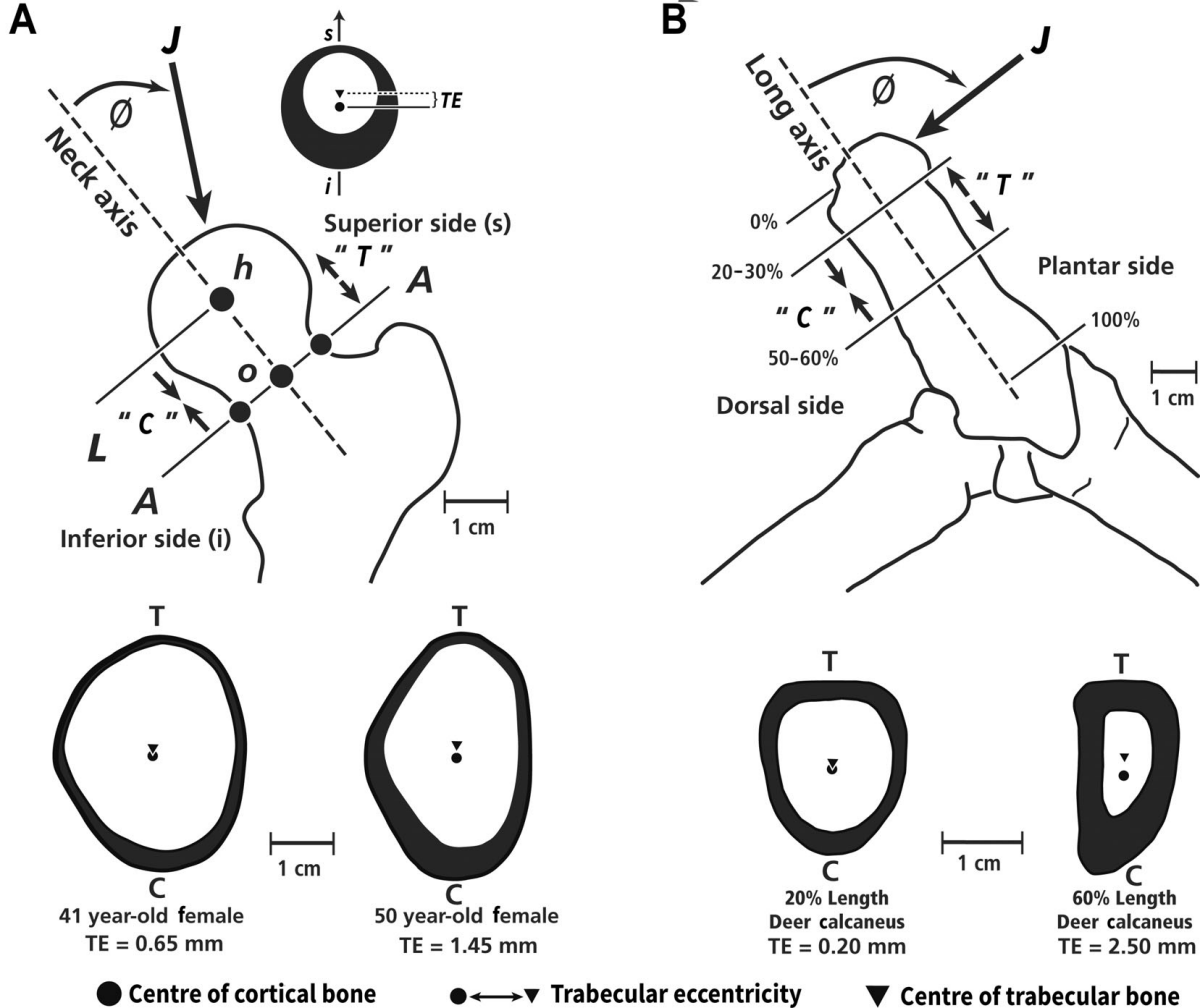
Trabecular eccentricity in adult human proximal femur (A) and adult deer calcaneus (B). (A) Diagrammatic depiction of a transverse cross section of an adult human proximal femur taken at the narrowest point of the femoral neck (*A–A* in the longitudinal section); *o* = centre of neck section, *h* = centre of head, *J* = joint load, TE = trabecular eccentricity, *C* = compression region, *T* = tension region. Bottom images are tracings of actual femoral neck cross sections. (B) Drawing of the deer ankle (talocrural joint), inverted to show the ‘plantar/tension cortex’ towards the top to mirror the ‘superior/tension cortex’ of the human femoral neck in A. Bottom images show transverse sections of the deer calcaneus also inverted to correspond with the orientation in A. Reproduced from Skedros *et al*. (2023) with permission of John G. Skedros.

A beam‐like bone region subjected to habitual unidirectional bending will have opposing regions that experience net tension and net compression (Figs 1 and 3). Conventional understanding of bone functional morphology and adaptation argues that the ‘compression side’ will be preferentially augmented, often through increased cortical thickness, because bone tissue is inherently stronger and more fatigue‐resistant in compression than tension (Reilly, Currey & Goodship, 1997; Boyce *et al*., 1998; Reilly & Currey, 1999; Skedros *et al*., 2006a; Diab & Vashishth, 2007). The thicker and more highly mineralized dorsal (compression) cortex of deer and sheep calcanei exemplifies this concept – these dual modifications likely enhance the strength of the bone in the loading mode to which it is best suited (Skedros *et al*., 1994a, 2007a). This conventional idea contrasts with the ‘trabecular eccentricity’ concept, which posits that optimal function of bone regions arises from a synergistic relationship between trabecular bone distribution and structural/material differences of the opposing cortices in net tension *versus* net compression. This concept was originally introduced in the context of habitual unidirectional (superior‐to‐inferior) bending of the human femoral neck (Fox & Keaveny, 2001, 2003). Trabecular eccentricity can be seen in cross sections of bones habitually subjected to bending and is characterized by the medullary area being filled with trabecular bone and asymmetric cortical thickness in the plane of habitual bending. This relationship is shown in Fig. 16 from a recent study comparing fracture risk factors (i.e. safety factors) in the context of the habitual load history between the deer calcaneus and the human femoral neck (Skedros *et al*., 2023). The artiodactyl calcaneus model is a useful comparison because its low‐complexity loading conditions help eliminate confounding influences in the more complex loading of the human femoral neck (Sinclair *et al*., 2013) (see Table 7). Note that the visual similarity between the arched trabecular patterns in artiodactyl calcanei and the upper human femur might falsely imply a similar load history (Fig. 14).

Estimating the mechanical benefit of trabecular eccentricity in bone regions under habitual unidirectional bending requires various input data, including mechanical properties for the trabecular bone and for the adjacent cortices [i.e. elastic modulus (tissue/material stiffness), yield stress, and ultimate stress]. These mechanical property data for dorsal and plantar cortices of the adult mule deer calcaneus are provided in Table 8. A main finding of our trabecular eccentricity analysis of the deer calcaneus was similar to the analytical studies of the human femoral neck (Fox & Keaveny, 2001, 2003): opposing cortices are adapted synergistically, reducing fracture risk for the entire region, rather than for each cortex in isolation. This is contrary to the conventional idea that biomechanical optimization occurs quasi‐independently in each cortex for its prevalent/predominant loading mode – compression in the dorsal cortex and tension in the plantar cortex (Skedros *et al*., 1994b, 1997, 2001). Thus, optimization of each cortical region does not occur; rather, synergism of the mechanical properties and distribution of trabecular and cortical bone optimizes the entire shaft region. Hence, if viewed in isolation, the adapted state of each opposing cortex (expressed as fracture risk reduction) appears sub‐optimal.

In their trabecular eccentricity analysis, Fox & Keaveny (2001, 2003) used a two‐dimensional analysis of loading of the upper femur without consideration of influences of muscles and ligaments, which can significantly affect the strain distributions in this region (Rudman, Aspden & Meakin, 2006; Nawathe *et al*., 2015; Kersh *et al*., 2018; Deng, Gillette & Derrick, 2021). In addition to considering the influence of trabecular eccentricity alone in optimizing the functional loading of the mule deer calcaneus, we also explored the load‐sharing influences of the relevant soft‐tissue structures: the nearby PL and SDFT (Fig. 1). These *ex vivo* strain measurements suggest that the plantar cortex primarily experiences tension during the majority of the simulated loading phase. Our sequential cutting experiment revealed that the intact PL reduces tension strain on the plantar cortex by ~35% with 50 kg load and ~49% with 100 kg load (Table 5) (Skedros *et al*., 2019). Additionally, that study revealed that the SDFT, when intact, reduces tension strain on the plantar cortex by ~52% with 50 kg load and ~29% with 100 kg load. The compression strains on the dorsal cortex under 50 kg load are also reduced, but to a notably lesser extent. Relatively modest strain reduction on the dorsal/compression cortex also occurs after separation of the PL from the plantar cortex (Table 5). These findings emphasize that the PL and SDFT influence strains in the relatively distant dorsal cortex.

These data show that when the PL and SDFT are cut, the tension strains on the plantar cortex nearly double (Table 5), resulting in substantial increases in fracture risk reflected by reductions in safety factors to levels of 2.1 or lower (Pattin, Caler & Carter, 1996; Skedros *et al*., 2003a). By contrast, because the compression strains in the dorsal cortex are influenced relatively less when the PL and SDFT are cut, the safety factors of the dorsal cortex remain much higher (above 6), which is a safer range for normal and most accidental load conditions (Biewener, 1993; Skedros *et al*., 2003a). Therefore, these data show that while the PL and SDFT reduce strain across the entire shaft, their combined influence is about four times greater on the plantar cortex. Hence, the plantar cortex can be considered a ‘fibro‐osseous cortex’, reflecting the biomechanical contributions of these soft tissue structures, which does not occur along the dorsal cortex due to the absence of adjacent/nearby load‐sharing structures.

Artiodactyl calcanei could be useful experimental models for advancing current understanding of how trabecular bone in general, and variations in specific trabecular bone architectural characteristics (e.g. plates *versus* rods, degree of anisotropy, bone volume fraction, connectivity, and trabecular length and number) help to transmit loads. To understand better the importance of trabecular bone in the general function of the artiodactyl calcaneal shaft, experiments could be performed where the calcaneal shaft, with multiple strain gauges attached, is loaded in bending before and after trabecular bone removal. This could be accomplished by drilling into the proximal‐most aspect of the bone and removing trabecular bone from the shaft with a burr, similar to the *ex vivo* experiments conducted by Holzer *et al*. (2009) on human femoral necks. This methodology would allow for attachment of strain gauges on the endosteal surface inside the medullary region to measure strains directly on the deepest surfaces of the cortex. This would help the development of more robust finite element models, as strains beneath the surface of a bone ultimately must be directly measured to ensure model accuracy (Bay, 2001; Turunen *et al*., 2020; Amraish & Pahr, 2024).

### (3) Architectural and material adaptations in trabecular bone

**Fig. 17:**
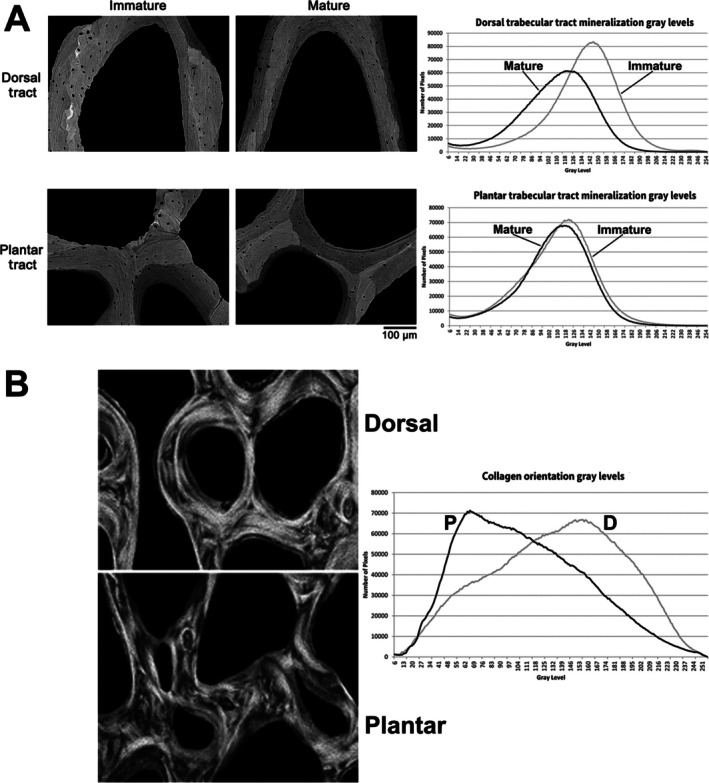
Microscopic images showing mineralization heterogeneity and predominant collagen fibre orientation (CFO) and heterogeneity in trabecular bone of deer calcanei. (A) Backscattered electron (BSE) images from transverse sections of the mid‐shaft region. Grey‐level profiles on right correspond to the images shown. Mineralization heterogeneity, typically considered beneficial for mitigating microdamage propagation, is quantified as the full width at half‐maximum (FWHM) of the main grey‐level peak in each profile. (B) Circular polarized light (CPL) images of thin (100 μm) transverse sections showing brightness (birefringence) differences between dorsal trabecular (D) and plantar trabecular (P) tracts of a mature deer calcaneus. Reproduced from Skedros *et al*. (2012b) with permission of John G. Skedros.

The artiodactyl calcaneus has been explored as a model for studying the adaptability of trabecular bone architecture, although only in a limited number of studies (Skerry & Lanyon, 1995; Skedros *et al*., 2012b; Sinclair *et al*., 2013). Many studies report data that strongly support the conventional view that trabecular bone adapts exclusively *via* architectural modifications (e.g. Turner, 1989; Ulrich *et al*., 1999; Keaveny *et al*., 2001; Barak, Lieberman & Hublin, 2011; Kivell, 2016; Hart *et al*., 2017) (see also Section VII.1). Therefore, it would be unexpected to find the presence of biomechanically significant material adaptations in trabecular bone in different regions of the same bone, even if subjected to simple unidirectional bending. However, this is precisely what we observed in the artiodactyl calcaneus, representing an extraordinary example of the capacity of trabecular bone to exhibit material‐level adaptations that likely enhance mechanical properties beyond what architectural characteristics alone can provide. In deer calcanei, we found statistically significant differences between the ‘compression trabecular tract’ and ‘tension trabecular tract’ at the level of the: (*i*) predominant collagen orientation of hemi‐osteons that mirrors the strain‐mode‐related CFO differences found in the secondary osteons of the dorsal and plantar cortices; and (*ii*) mineralization heterogeneity of their trabecular bone ‘packets’ (Skedros *et al*., 2012b, 2025), which are basic structural units (typically hemi‐osteons) within trabecular bone (Parfitt, 1979; Ciarelli *et al*., 2009; Smith, Schirer & Fazzalari, 2010; Lamarche *et al*., 2025) (Fig. 17). Differences between the dorsal and plantar trabecular tracts likely help accommodate differences in their strain environments, implying that strain‐mode‐related adaptation can occur within the matrix of individual trabeculae.

Lozupone (1985) used polarized light to examine CFO patterns in microscopic images of thin transverse sections of individual trabeculae in two regions of adult human calcanei. One region is believed to be primarily loaded in tension and the other in compression due to simple unidirectional bending presumed to occur during typical walking/running (Gierse, 1976; Giddings *et al*., 2000; Gefen & Seliktar, 2004; Bajraliu, Walley & Kwon, 2016; Saers, Ryan & Stock, 2020; Saers *et al*., 2022). However, Lozupone (1985) did not find the expected strain‐mode‐related CFO differences between these regions. This absence may reflect differences in locomotion patterns; human locomotion is highly variable, whereas deer typically employ a stereotyped para‐sagittal gait (Lingle, 1992; Ducharme & van Emmeril, 2018; Skedros *et al*., 2023). In a study of the material organization of trabecular bone of adult human femoral necks, von Kroge *et al*. (2022) reported a similar lack of differences in predominant CFO between the superior (‘tension’) and inferior (‘compression’) trabecular tracts (Fig. 14). Perhaps the absence of regional differences in CFO between the putative tension *versus* compression trabecular tracts of the human femoral neck may reflect the more complex loading of that region. In fact, von Kroge *et al*. (2022) suggested that shear stresses (indicative of increased complexity of loading) might be involved in creating the seemingly paradoxical CFO patterns in the opposing trabecular bone regions and nearby cortices of the femoral neck. These results favour the idea that the human calcaneus and femoral neck are in a higher complexity load category than the artiodactyl calcaneus (Table 7).

## VII. DIFFERENTIAL ADAPTABILITY OF CORTICAL *VERSUS* TRABECULAR BONE

### (1) The artiodactyl calcaneus as a ‘control bone’

We introduced the artiodactyl calcaneus model as a comparative ‘control bone’ for studies examining the interplay between trabecular and cortical bone. Sheep and deer calcanei also offer valuable opportunities for exploring specific architectural and material characteristics of trabecular bone that can enhance understanding of trabecular bone biomechanics and load‐history interpretation (Sinclair *et al*., 2013). The use of a ‘control bone’ is especially important in view of the growing body of studies that purport finding a ‘signal’ in trabecular bone structure that reflects habitual load direction or behaviour (e.g. climbing *versus* walking) (Mullender & Huiskes, 1995; Ryan & Shaw, 2012; Kivell, 2016; Barak, Sherratt & Lieberman, 2017; Kivell *et al*., 2018; Ryan *et al*., 2018). These studies suggest that specific architectural characteristics of trabecular bone can represent functional signals of joint loading that are useful for identifying differences in habitual load histories in bones of extant and extinct species (Ryan & Shaw, 2012, 2013; Barak *et al*., 2013; Sinclair *et al*., 2013; Matarazzo, 2015; Kivell *et al*., 2018; Georgiou *et al*., 2019; Komza & Skinner, 2019; Saers *et al*., 2022). Examples of the trabecular bone characteristics proposed to have these properties, either individually or synergistically, include: degree of anisotropy, predominant trabecular orientation, mean trabecular thickness, connectivity, prevalence of rods *versus* plates, and bone volume/total volume (BV/TV = bone volume fraction). One shortcoming of these studies is that, in most cases, the load histories within trabecular regions of the bone are poorly understood, and the strain distributions and magnitudes at the level of individual trabeculae are even less clear. Consequently, some reported associations between habitual load history and specific trabecular architectural characteristics may not represent causal structure–function relationships or are not strong correlates of structure–function relationships. Sinclair *et al*. (2013) failed to find a ‘signal’ in trabecular bone structure that reflected habitual load history of sheep and deer calcanei, despite their simple loading. However, the latter study did not account for the marked differences in locomotor activities between these two artiodactyl species (see Section II.3) that might have influenced these results.

Nevertheless, some experimental studies have advanced our understanding of the mechanisms involved during development of trabecular bone architecture. Experimental models have been introduced that have potential for determining more directly how strains are distributed within a bone's trabecular lattice (Biewener *et al*., 1996; Pontzer *et al*., 2006; Barak *et al*., 2011; Yang *et al*., 2013; Kivell, 2016). Studies that aim to interpret a bone's load history in the absence of *in vivo* strain data should consider how trabecular and cortical bone might work synergistically, as demonstrated by Saers *et al*. (2021) and foreshadowed by trabecular eccentricity studies discussed in Section VI.2. Experimental studies show that cortical and trabecular bone respond differently (in terms of osteogenic response) to applied strains, and that trabecular bone is relatively more responsive to low‐amplitude strains. This, together with other differences in the responsiveness of cortical and trabecular bone before and after skeletal maturity, must be considered in studies aimed at understanding more fully the functional adaptation of the artiodactyl calcaneus. Additional discussion of differences in responsiveness of cortical and trabecular bone to applied loads can be found in Saers *et al*. (2021) and Barak (2024).

Bone mineral density (BMD, mg/cm^3^) includes non‐bone porosity and is the most studied material parameter in investigations of the functional adaptation and adaptability of trabecular bone. It is important to emphasize that trabecular BMD represents the bulk amount of trabecular bone in a unit volume. While BMD can be influenced by true tissue density (i.e. mineralization of the bone material/tissue), this is typically considered negligible because of the low variation in true tissue density in trabecular bone across various anatomical locations in adult and growing animals (Hernandez *et al*., 2001; Nazarian *et al*., 2008; Skedros *et al*., 2012b). Additionally, in trabecular bone there is little to no correlation between BMD and tissue density (Hernandez *et al*., 2001). However, a biomechanically important interaction between BMD and tissue density cannot be discounted in the artiodactyl calcaneus. Statistically significant differences in true tissue density (expressed as ash mineral content) have been described in the dorsal *versus* plantar trabecular bone regions in growing and adult deer calcanei, in addition to differences in trabecular architectural characteristics (Skedros *et al*., 2012b; Sinclair *et al*., 2013). Studies of the potential mechanical relevance of these material‐level variations are needed.

### (2) Coupling of material/tissue‐level and structural/architectural characteristics

Studies of human trabecular bone have revealed associations between specific architectural (structural) characteristics and material characteristics. For example, a study of trabecular bone in human lumbar vertebrae showed that the size and prevalence of hemi‐osteonal ‘packets’, along with their associated cement line length and density, were strongly correlated with trabecular bone volume fraction, structure model index (a measure of rod *versus* plate‐like morphology), and compressive strength (Lamarche *et al*., 2025). In human trabecular bone from the proximal femur and lumbar vertebrae, differences in tissue mineral density have been reported between plate‐ and rod‐like trabeculae and among various trabecular orientations (Wang *et al*., 2015). Additionally, microcrack density has been reported to correlate with various structural characteristics (bone volume fraction, trabecular number, structure model index, and trabecular separation) (Arlot *et al*., 2008). However, these studies did not examine regions with potentially distinct strain modes or magnitudes. Differences in histomorphology and mineralization between the dorsal and plantar tracts of deer calcanei (Skedros *et al*., 2012b; J. G. Skedros, J. T. Cronin, C. S. Mears & B. W. Richards, in preparation) might be correlated with architectural characteristics in ways that have previously gone undetected. The few studies that have explored such structural–material coupling are hampered by limited material‐level characterizations (e.g. focused only on mineralization), unclear load history, or age‐related effects that are difficult to control (e.g. use of human bones) (Torres *et al*., 2016; Yu *et al*., 2021; Lamarche *et al*., 2025).

### (3) Manifestation of load history variations

There are strong opinions that trabecular bone can manifest load history more readily than cortical bone, despite a lack of evidence from *in vivo* strain data in trabecular bone (Kivell, 2016; Barak, 2024). A ‘control bone’ where *in vivo* strains can be measured within the trabecular bone would be invaluable for examining this assertion. Furthermore, when compared to cortical bone, trabecular bone adaptation can be confounded more substantially by disuse‐related bone loss in addition to stimuli that might not be strongly associated with ambient strains, including allometric and genetic influences, and calcium homeostasis (Swartz, Parker & Huo, 1998; Squire *et al*., 2008; Doube *et al*., 2011; Gosman, Stout & Larsen, 2011; Wysocki & Tseng, 2018). Currently, finite element analyses are the best way to estimate magnitudes and strain distributions throughout the trabecular lattice and in the subsurface cortical bone (Tsubota *et al*., 2009; Oftadeh *et al*., 2015; Ruffoni & van Lenthe, 2017; Turunen *et al*., 2020). Digital image correction (DIC) of high‐resolution full‐field strain maps is currently the best technology for measuring strains on individual trabeculae within the trabecular lattice (Turunen *et al*., 2020; Amraish & Pahr, 2024). This methodology is imperative for the further development of this experimental model and should be a focus of future studies in artiodactyl calcanei.

### (4) Mechanisms of bone growth modelling

Trabecular bone modelling during growth (i.e. ‘mini modelling’ of the trabeculae) may influence the temporal and spatial organization of trabecular bone in ways that are poorly understood (Jee, Tian & Setterberg, 2007; Wood *et al*., 2019). For instance, it is unclear whether CFO patterns within an individual trabeculum (or between trabecular regions/tracts) are retained from earlier phases of postnatal ontogeny, when trabecular volume fraction changes are likely more important than the creation of trabecular architectural adaptations (Tanck *et al*., 2001). The artiodactyl calcaneus is a compelling model in this early ontogeny context, as it exhibits clear, functionally relevant architectural changes in the prenatal period (i.e. arched trabecular patterns resembling stress trajectories of simple bending). Specifically, arched trabecular patterns, as shown in Figs 1 and 14 are present in fetal development in deer and sheep calcanei (Skedros *et al*., 2004). Whether these arched patterns reflect *in utero* loading from limb motion (i.e. tension *versus* compression stress trajectories) or are the result of programmed development remains unknown.

Goodship & Lanyon [reported by Skerry (2000)] observed that transection of the Achilles tendon of a fetal sheep *via in utero* surgery disrupted the formation of the typical arched patterns seen at birth, instead resulting in disorganized trabeculae. This compelling observation was not subsequently pursued with additional experimentation (Lance Lanyon, personal communication). Today, intrauterine surgery is often performed on fetal sheep for studying the pathogenesis and treatments of conditions such as cardiothoracic pathology and myelomeningocele in humans (Turley *et al*., 1982; Wallen *et al*., 1994; Baumgarten & Flake, 2019; Danzer *et al*., 2020; Lussier *et al*., 2025). Future experiments could couple *in utero* sciatic neurectomy aimed at reducing neurotrophic effects on the growing skeleton (Lanyon, 1980) with Achilles tenotomy. Since few studies have considered the embryological development of artiodactyl calcanei, the relative influences of extra‐genetic (e.g. functional loading) *versus* genetic influences in shaping their structural and material characteristics remain unclear (Skedros *et al*., 2007a). Ossification of the artiodactyl calcaneus (showing two centres of ossification) has been reported in a radiographic study by Dhingra & Tyagi (1970), but no details of trabecular architecture were provided. Additional information is mostly limited to brief, decades‐old mentions in obscure or non‐English journals. This area of research is very deficient.

Future studies incorporating in‐depth examination of the collagen matrix composition/organization (e.g. after demineralization) and advanced analysis techniques (Smith *et al*., 2010; Georgiadis, Muller & Schneider, 2016; Stockhausen *et al*., 2021) will likely reveal additional manifestations of regional load‐history‐related material adaptations of the trabecular bone of sheep and deer calcanei across ontogeny, perhaps even at the molecular levels of tissue organization/composition (Saito & Marumo, 2010; Granke *et al*., 2013; Reznikov *et al*., 2015, 2018; Xie *et al*., 2018; Skedros *et al*., 2024b).

## VIII. FUTURE STUDIES

**Fig. 18:**
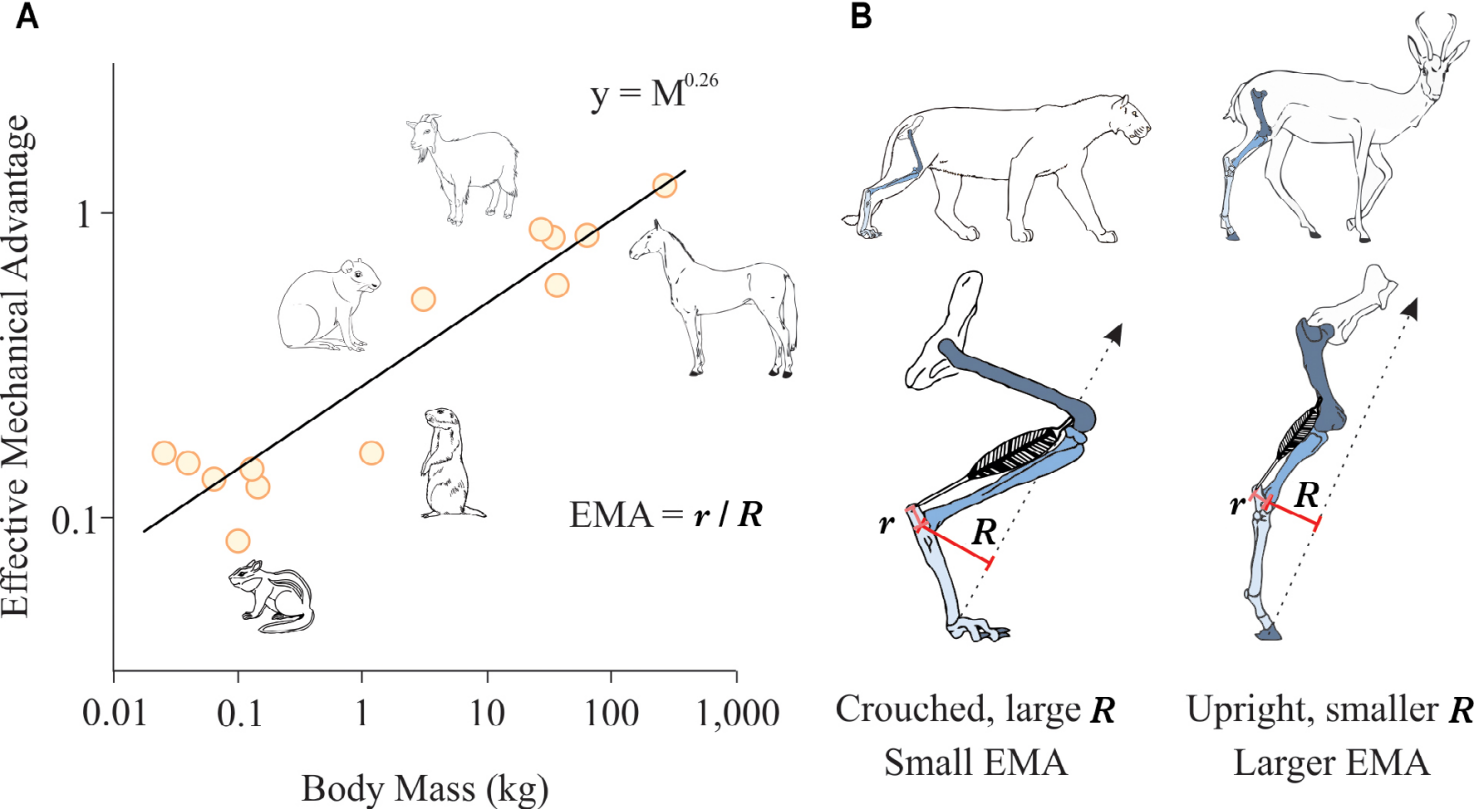
Effect of body size and posture on limb effective mechanical advantage (EMA). EMA is the ratio of extensor muscle moment arm (*r*) to the resultant three‐dimensional (3D) ground reaction force moment arm (*R*). (A) Hindlimb EMA scaling for mammals. Data from Biewener (1989, 2005) shows that EMA scales with *M* ^0.26^ (*M* = body mass). (B) Effect of hindlimb posture on limb EMA shows that crouched animals have a lower limb EMA than upright animals like artiodactyls. The dashed black arrows represent the ground reaction force vector. Reproduced from Dick & Clemente (2017) (open access under terms of Creative Commons Attribution License).

In addition to sheep and deer, calcanei of other animals (e.g. other artiodactyls, potoroos, canines, felids, rabbits, horses and pigs) are reported to exhibit arched trabecular patterns when viewed in lateral radiographs or thin cross sections, likely reflecting habitual unidirectional bending (Geiser & Trueta, 1958; Vander Sloten & Van der Perre, 1989; Mgasa & Arnbjerg, 1993; Biewener *et al*., 1996; Kofler & Sullmann, 2021; Olgun Erdikmen *et al*., 2021; Şenol *et al*., 2022; Cottereau *et al*., 2023; Jing *et al*., 2024; Pinna, Tassani & Di Benedetto, 2024). These could prove useful for comparative analyses of strain‐mode‐related bone adaptation in additional basic and clinical contexts, including osteopenia/osteoporosis‐related research that is often conducted in small animal models (Geiser & Trueta, 1958; Leung *et al*., 2001). When comparing calcanei of artiodactyls with those of smaller animals (e.g. rabbits and potoroos), the biomechanical consequences of differences in limb posture should also be considered. Larger animals (like most artiodactyls) generally have increased effective mechanical advantage (EMA) in limb joints, which reduces stress during locomotion compared to the crouched postures of smaller animals (Fig. 18) (Biewener, 1990; Dick & Clemente, 2017).

### (1) Artiodactyl calcanei and the evaluation of stress fracture

There are traditionally three types of stress fractures: fatigue fractures due to overuse, insufficiency fractures due to bone fragility, and pathological fractures due to bone weakness involving tumours (Pentecost, Murray & Brindley, 1964; Oh *et al*., 2021). Fatigue fractures occur in normal bone that has been overused (e.g. in military personnel and athletes), whereas insufficiency fractures develop in fragile bone repeatedly subjected to low levels of stress during everyday physical activity (e.g. a femoral neck fracture in an elderly human from a ground‐level fall) (Oh *et al*., 2021). Racing greyhounds often experience tarsal stress/fatigue fractures that include the calcaneus (Devas, 1961; Boudrieau, Dee & Dee, 1984; Ost *et al*., 1987; Muir, Johnson & Ruaux‐Mason, 1999). Consequently, detailed structural and material analyses of calcanei from these animals could advance understanding of how naturally occurring bending‐related adaptations can become inadequate for mitigating the deleterious effects of highly repetitive, high‐magnitude bending stresses during racing.

In the greyhound calcaneus and in nearly all other bones where stress fractures occur in any species, little is known about how the antecedent/baseline material organization (i.e. histomorphology and histocomposition) influences the pathogenesis of stress fractures (Skedros *et al*., 2012a, 2024b; Richards *et al*., 2024). This could be studied by comparing data from normal bones to those with stress fractures. For example, post‐mortem microanalysis or non‐destructive high‐resolution CT imaging of the greyhound calcaneus or other bones could reveal potential correlations with: (*i*) woven bone formation (which might indicate a healing response); (*ii*) lamellar bone formation that might reflect very high strains (which might occur without fracture) (Turner *et al*., 1994) (see Section V); and/or (*iii*) other regional variations in bone histomorphology. As described by Nunamaker (2001), knowledge of regional variations in bone histomorphology of the third metacarpal (MC3) of racehorses has been instrumental in devising training programs that reduce the incidence of stress fractures, likely by minimizing the deleterious tissue‐level effects caused by strain reversals that occur when horses transition from typical galloping to their fastest racing speeds (Nunamaker, 2001; Skedros *et al*., 2024b). More specifically, equine MC3s have been studied in terms of how regional variations in material characteristics (e.g. the amount of osteonal remodelling, population densities of osteon collagen/lamellar morphotypes, and collagen cross‐links): (*i*) accommodate typical strain distributions of natural loading (i.e. not racing) because the bone has adapted histologically to that load history (e.g. tension‐specific adaptation on one side of the bone and compression‐specific adaptation on the other) (Table 2); and (*ii*) fail to accommodate the stark differences in mechanical requirements in regions where strain‐mode reversals occur because the neutral axis shifts/rotates at the fastest gait speeds (and deleterious amounts of microdamage accumulate because the bone is not histologically adapted for the change in strain environment) (Reilly & Currey, 1999, 2000; Skedros *et al*., 2024b).

Natural models like the equine MC3 and greyhound calcaneus, however, are limited in establishing cause‐and‐effect relationships due to poor control of key variables. As emphasized by Burr (2001), experimental models, such as long bones in rabbits, offer a more effective approach for identifying causal factors in stress fracture pathogenesis. In this case, it remains critical that natural regional variations in histomorphology/histocomposition (as listed in Tables 1 and 2) of the specific bone model are well characterized to determine how pre‐lesion/pre‐injury tissue organization might influence stress fracture pathogenesis. Muir *et al*. (1999) quantified the regional presence, numerical density, and morphology of *in vivo* microdamage in the central tarsal bone of greyhounds with and without stress fracture. Similar analysis of the greyhound calcaneus could be highly informative in animals with and without a stress fracture of that bone, especially in comparing histomorphology/histocompositional differences between cortical regions (e.g. dorsal *versus* plantar). Such data obtained from sub‐adult and adult sheep and deer calcanei in normal conditions could be used for comparative analyses in studies of greyhound calcanei and stress‐fracture‐prone bones in other species. For example, the comprehensive data obtained from deer and sheep calcanei (Table 2) have highlighted how similar robust analyses of regional variations are conspicuously lacking in the human femoral neck, even in normal conditions (Skedros *et al*., 2023). Such data would also be useful for advancing understanding of the pathogenesis of stress and some fragility fractures in the human femoral neck, as these fractures are often considered either tension‐ or compression‐dominant (Mayhew *et al*., 2005; Shaw *et al*., 2022; Richards *et al*., 2024).

In stress fractures of the greyhound calcaneus, the relative contribution of tension *versus* compression is likely variable and may be correlated with fracture pathogenesis. For instance, greyhound calcaneus fractures that occur independently of central tarsal fractures are thought to result from extreme tension on the plantar aspect of the bone (Ost *et al*., 1987; Perry *et al*., 2017). This suggests a dorsal‐direction shift in the neutral axis, as has been shown following deficiency/injury to the plantar ligament of the deer calcaneus (Skedros *et al*., 2019). Consequently, it is possible that, at racing speeds, certain regions of the calcaneus and/or other tarsal bones of the greyhound, like the equine MC3, experience deleterious strain‐mode reversals. In this hypothetical scenario, these reversals may occur with sufficient frequency and intensity during racing and race training to cause excessive microdamage in regions that are not adapted for the ‘new’ predominant strain mode. As emphasized in Section II.5, consideration of the often‐overlooked ‘third strain mode’ – shear – is also crucial when considering the details of stress fracture mechanobiology/etiology.

### (2) ‘Geometric morphometrics’ and related methods

**Fig. 19:**
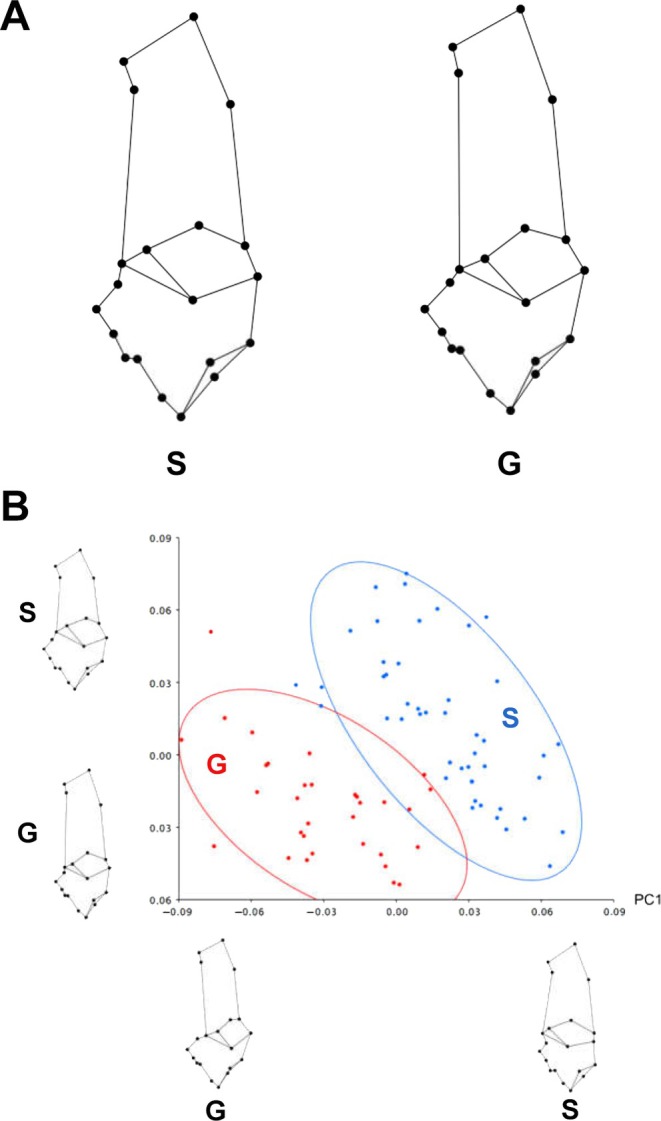
(A) Landmark locations from medial views of a sheep (S) and goat (G) calcaneus, with average shapes depicted. (B) Scatter plot of the principal component (PC) analysis based on the weight matrix of residuals from the multivariate regression with the corresponding extreme calcaneus shapes of every axis. 90% confidence ellipses are shown: red = goat; blue = sheep. Reproduced from Lloveras *et al*. (2022) (open access under terms of Creative Commons Attribution License).

Artiodactyl calcanei have been used in studies employing ‘geometric morphometrics’, an approach that analyses object shape using Cartesian landmarks capable of representing morphologically distinct shape variables (Fig. 19) (Curran, 2015; Pantinople *et al*., 2017; Dunn & Avery, 2020; Gruwier & Kovarovic, 2022; Lloveras *et al*., 2022; Parés‐Casanova *et al*., 2023). These studies are typically done in two dimensions and are generally more useful than linear measurements, particularly for understanding a bone's shape (especially joint surfaces). However, linear measures remain useful for relatively gross visualizations of bone length and curvature (Pantinople *et al*., 2017; Barr, 2020). Barr (2020) used such data to suggest that the longer calcaneal tubers (shaft portion) may reflect the importance of stotting behaviour for predator avoidance (Caro, 1986) in some species. The length of the distal calcaneus has been correlated with gait speed in cursorial terrestrial mammals (Christiansen, 2002) and leaping ability among prosimians such as lemurs (Boyer *et al*., 2013). Because deer and sheep calcanei are dense and taphonomically durable (Behrensmeyer, 1975; Borrero, 1990), they are frequently used in geometric morphometric studies aimed at understanding how ‘paleoenvironmental’ mechanical and non‐mechanical factors influence bone morphology (Curran, 2012, 2015; Gruwier & Kovarovic, 2022).

## IX. CONCLUSIONS

(1) Sheep and deer calcanei are important models for studying cortical and trabecular bone adaptation because they are amenable to direct strain measurement (they lack surrounding muscles), experience relatively simple/unidirectional bending, exhibit extensive osteon remodelling, and have the largest reported regional variations in mineralization and other histological characteristics.
(2) We found little support for the McMahon *et al*. (1995) challenge to the simplified concept of the habitual loading of the sheep calcaneus as a ‘tension/compression bone’. The new suggestion that the artiodactyl calcaneus might be viewed more accurately as a ‘tension–shear/compression bone’ (i.e. plantar/dorsal) warrants additional evaluation, especially in view of the paramount significance of the shear‐resistance priority hypothesis in many bone adaptation studies.
(3) Evaluation of the artiodactyl calcaneus model in the context of ‘trabecular eccentricity’ demonstrates how it can be used for evaluating similarly loaded bones and bone regions (e.g. the human femoral neck).
(4) *In vivo* strain data coupled with finite element analyses of the artiodactyl calcaneus model are needed to elucidate additional details of the biomechanical functioning and synergism between trabecular and cortical bone. Mechanical testing of trabecular bone specimens from the dorsal and plantar regions will be especially important for developing robust finite element models of sheep and deer calcanei. These studies will help advance understanding of the strengths and limitations of using these bones as natural and experimental models for investigating general and specific questions in bone adaptation, within a species, between related species, and in broader comparative contexts.
(5) Comparative studies across various bones and species are needed to examine the cellular mechanisms and mechanical consequences that explain the correlations of structural and material characteristics with the strain environment. These are especially needed during common and less‐common gait‐related activities and across ontogenetic stages from fetal development to birth through skeletal maturity to advanced age. The artiodactyl calcaneus model has great potential for these investigations and can serve as a ‘control bone’ in various research contexts.

## Supporting information


**Video S1.** Z‐stack microcomputed tomography (micro‐CT) video showing vascular pores from the dorsal cortex of an adult mule deer calcaneus at mid‐shaft. This video (and also Video S2) was constructed in a cylindrical region, as reported by (Skedros *et al*., 2014). Relative to the long axis of the cylindrical region, the pores are oriented obliquely. The cylindrical region measures 1.9 mm in diameter (transverse to the long axis of the shaft). The pores in this video are smaller (mean diameter 21 μm), more numerous, and have a lower degree of anisotropy (DA, mean 0.73) than those in the plantar cortex (mean diameter 26 μm and 0.76 mean DA). This cylindrical region was examined using a Skyscan™ 1172 scanner (Bruker Micro‐CT, Kontich, Belgium) at 78 kVp and 125 μA with a 0.5 mm aluminum filter. The images were rotated through 180° in 0.1° steps resulting in 1800 projections each involving 1.7 s exposures with 3‐exposure averaging to improve signal to noise ratio. The resulting data sets had an isotropic voxel size of 2μm^3^ [<5 μm X‐ray source spot size; 8.83 μm camera (physical) pixel size].

**Video S2.** Z‐stack microcomputed tomography (micro‐CT) video showing vascular pores from the plantar cortex of an adult mule deer calcaneus at mid‐shaft. This video (and also Video S1) was constructed in a cylindrical region, as reported by (Skedros *et al*., 2014). Relative to the long axis of the cylindrical region, the pores are oriented obliquely. The cylindrical region measures 1.9 mm in diameter (transverse to the long axis of the shaft). See legend for Video S1 for: (*i*) comparisons between the pores in the dorsal *vs*. plantar regions, and (*ii*) details of the Micro‐CT scanner and parameters used to create this video.
